# Supplementary figures and images for: Daptomycin treatment impacts resistance in off-target populations of vancomycin-resistant Enterococcus faecium
Source: PLoS Biol. 2020 Dec 17;18(12):e3000987. doi: 10.1371/journal.pbio.3000987 (PMC7775125; doi:10.1371/journal.pbio.3000987)

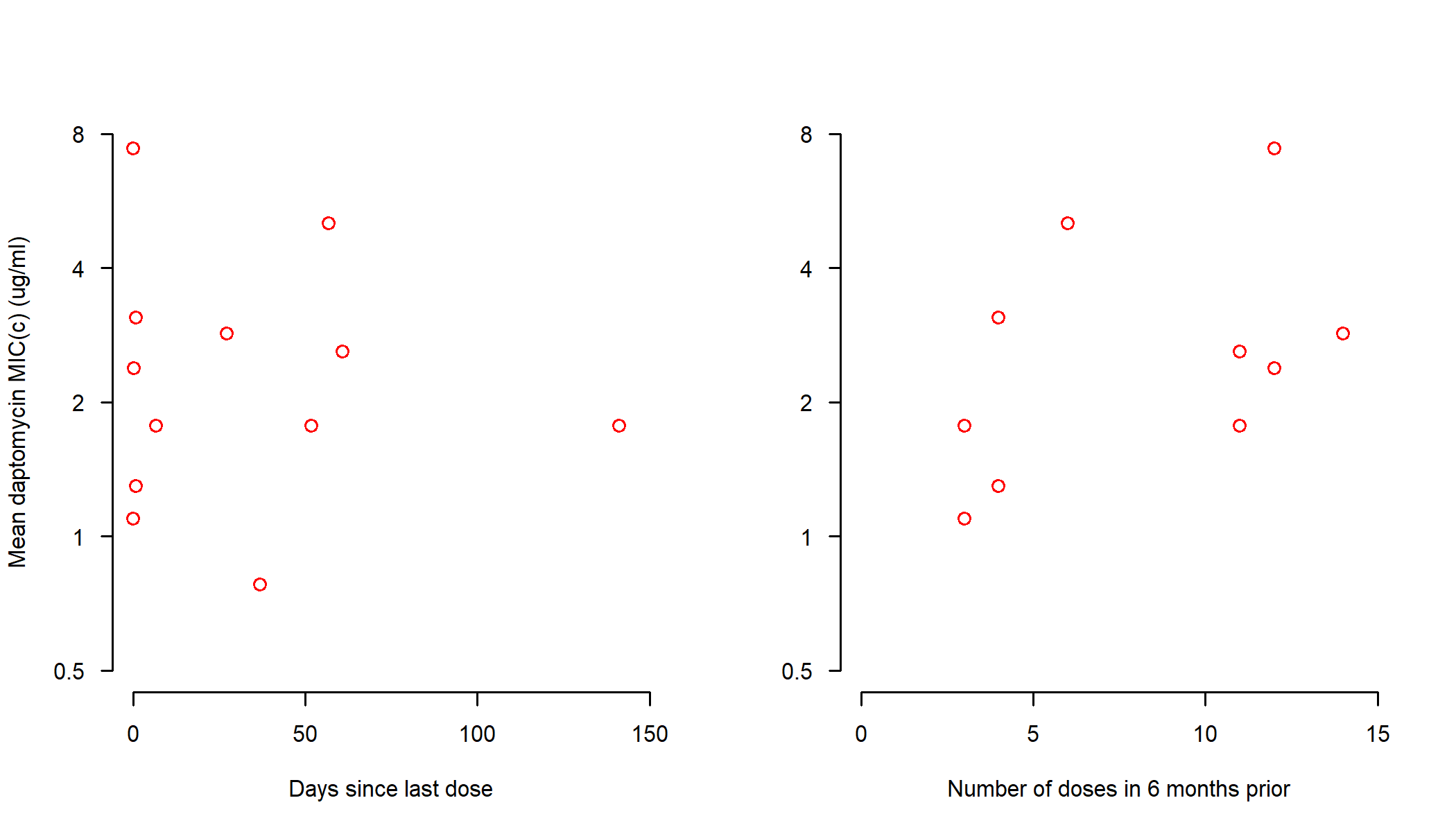

Supplement: S1 Fig — Mean daptomycin MICC per patient by (left) the number of days since the last dose and (right) the total number of daptomycin doses in the 6 months prior to the index sample. For underlying data see S1 Data. (TIF) [file pbio.3000987.s001.tif]

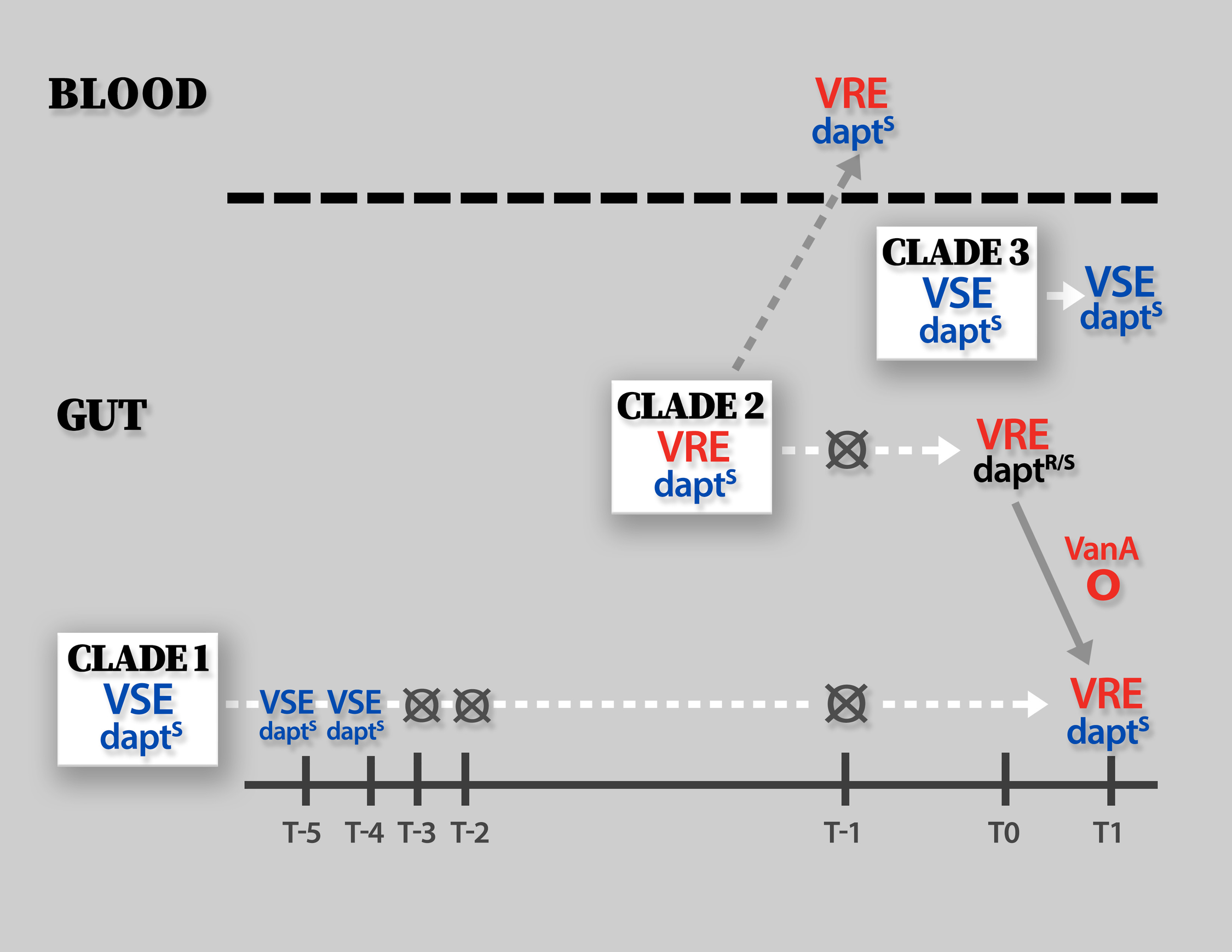

Supplement: S3 Fig — Based on the phylogenies in Fig 7, isolates from Patient 150 have been assigned to 1 of 3 clades. Clades were detected in sampling when they are noted above a time point and not detected if there is a gray ⊗. While Clade 1 was not detected in samples between T-3 and T0, it appears to have persisted through to T1, where it has acquired a VanA plasmid from Clade 2. Clade 2 was first isolated from the blood stream; however it is likely that this came from an undetected gastrointestinal population. (TIF) [file pbio.3000987.s003.tif]
