# Supplementary material for: Daptomycin treatment impacts resistance in off-target populations of vancomycin-resistant Enterococcus faecium
Source: PLoS Biol. 2020 Dec 17;18(12):e3000987. doi: 10.1371/journal.pbio.3000987 (PMC7775125; doi:10.1371/journal.pbio.3000987)
Supplement: S2 Fig — Time-series for each patient showing patient admission periods, drug doses, and resistance of E. faecium clones isolated from screening swabs and all Enterococcus blood stream infections. Patient admission periods are shown as gray blocks, and individual doses of vancomycin (purple), linezolid (yellow), and daptomycin (red) are detailed in the bars at the top of the plot. Circles show daptomycin resistance (MICC) for 10 clones per sample. Each circle is the mean of 2 replicates with black circles denoting VR E. faecium and blue circles denoting VS E. faecium. Diamonds are isolates from VR E. faecium (purple), VS E. faecium (blue), and VS E. faecalis (gray) blood stream infections. Panel A–Patients 20, 22, 92, and 96; Panel B–Patients 101, 103, 104, 157, 161, and 378; Panel C–Patients 4, 29, 32, 54, 64, and 84; and Panel D–Patients 86, 87, 150, 233, 237, and 239. For underlying data see S1 Data. (PDF) [file pbio.3000987.s002.pdf]

A

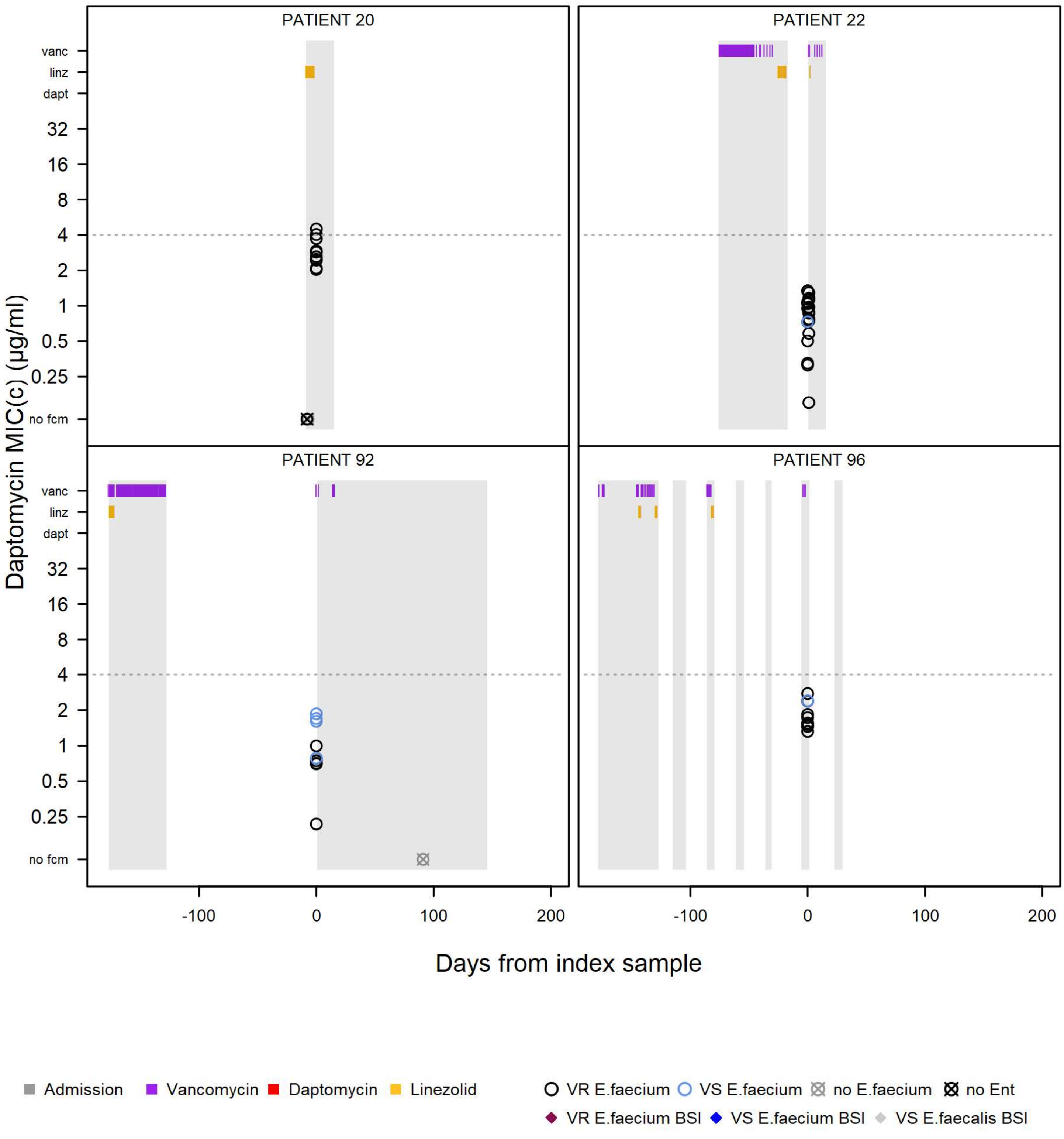
PATIENT 20
PATIENT 22
PATIENT 92
PATIENT 96
vanc
linz
dapt
32
16
8
4
2
1
0.5
0.25
no fcm
-100
0
100
200
Daptomycin MIC(c) (μg/ml)
Days from index sample
Admission
Vancomycin
Daptomycin
Linezolid
VR E.faecium
VS E.faecium
no E.faecium
no Ent
VR E.faecium BSI
VS E.faecium BSI
VS E.faecalis BSI

**B**

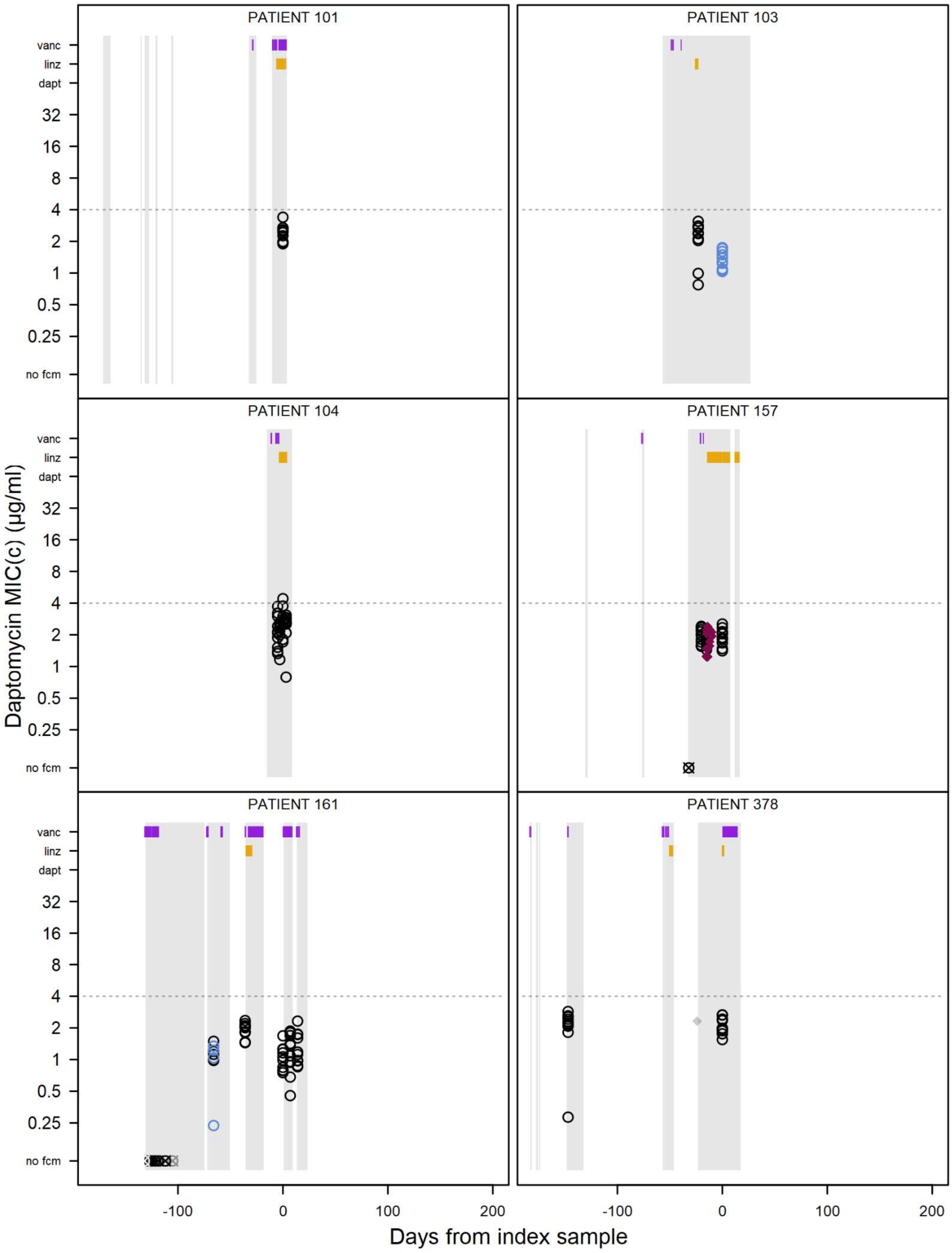
PATIENT 101
PATIENT 103
PATIENT 104
PATIENT 157
PATIENT 161
PATIENT 378
vanc
linz
dapt
32
16
8
4
2
1
0.5
0.25
no fcm
Daptomycin MIC(c) (µg/ml)
-100
0
100
200
Days from index sample

C

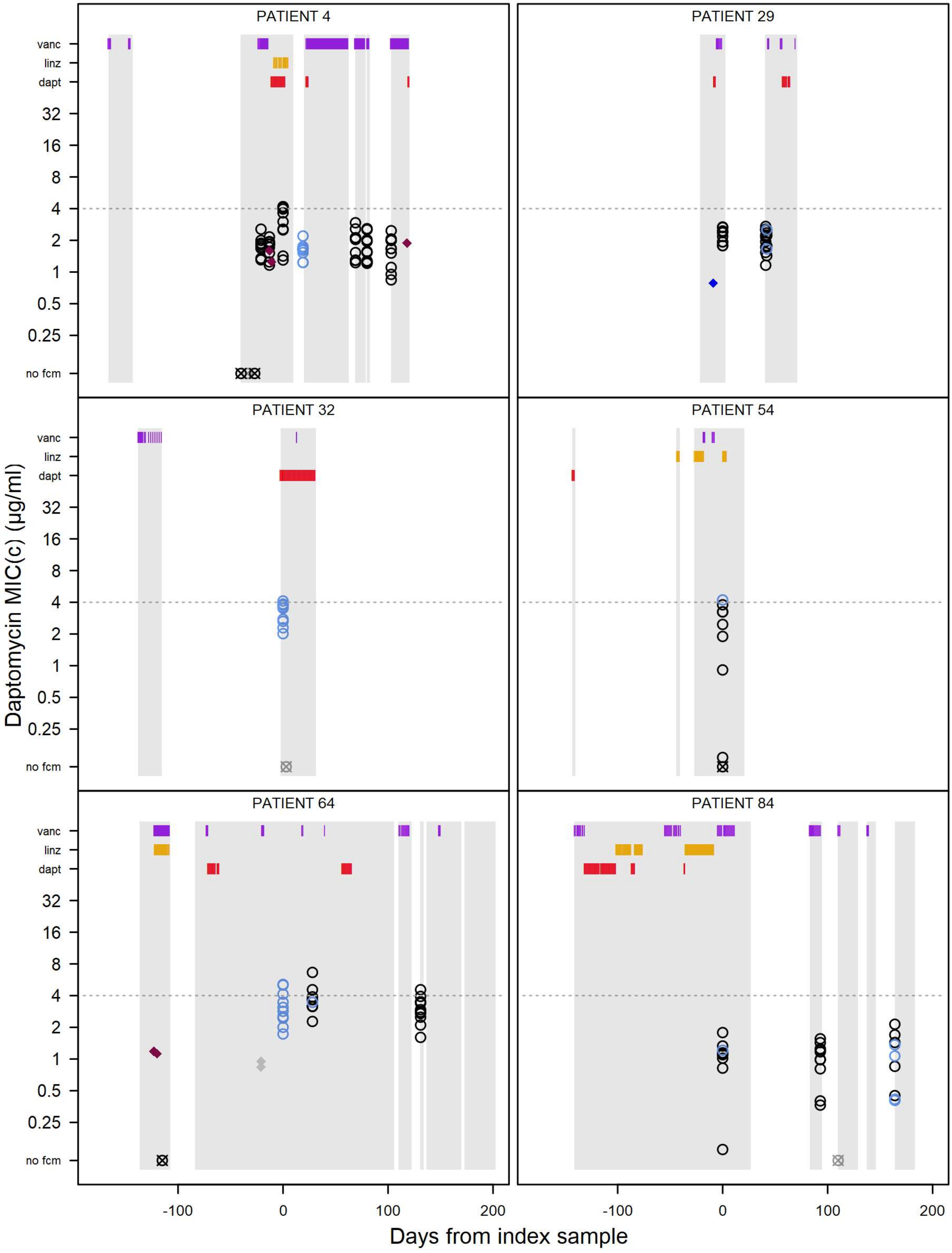
PATIENT 4
PATIENT 29
PATIENT 32
PATIENT 54
PATIENT 64
PATIENT 84
vanc
linz
dapt
32
16
8
4
2
1
0.5
0.25
no fcm
Daptomycin MIC(c) (µg/ml)
-100
0
100
200
Days from index sample

D

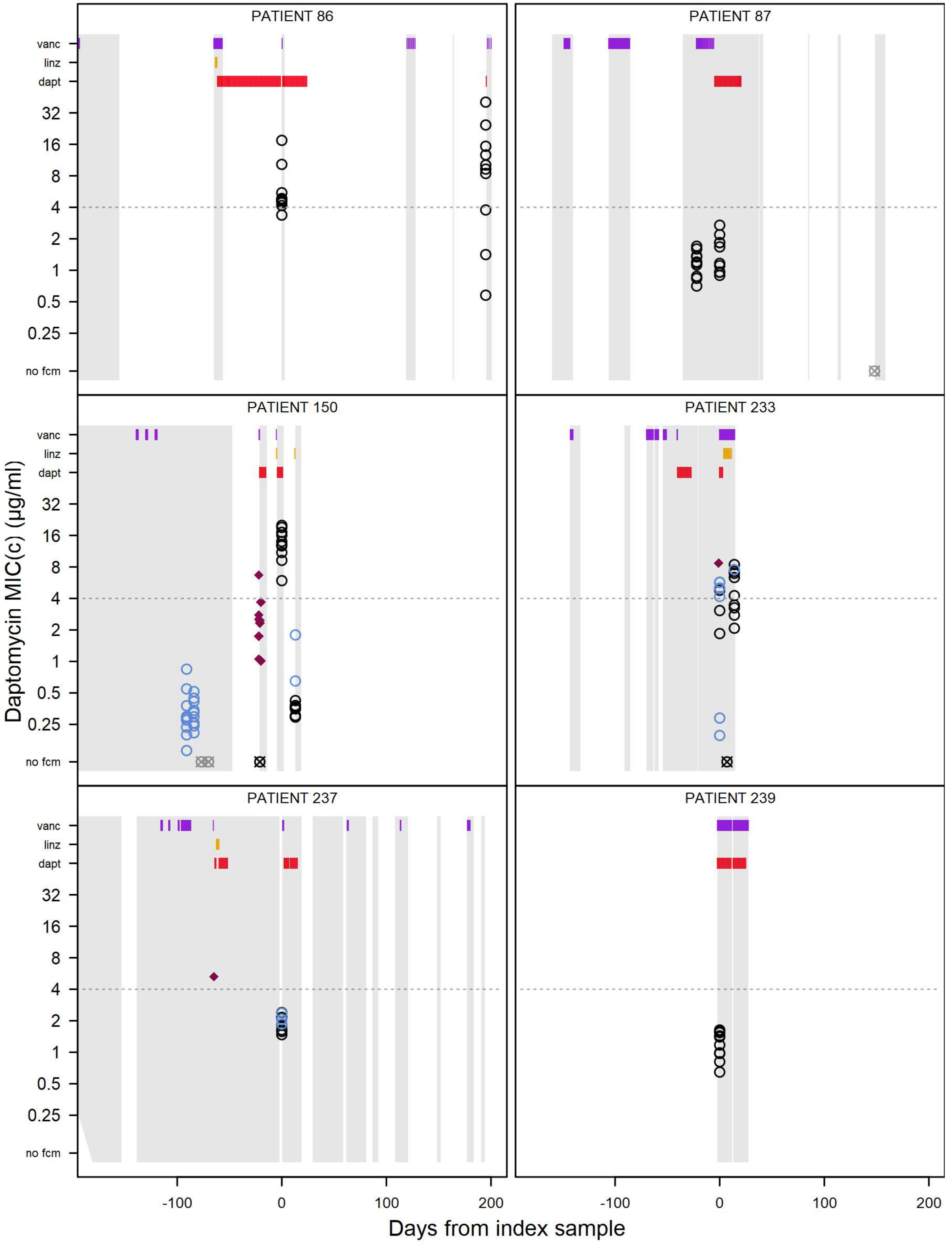
PATIENT 86
PATIENT 87
PATIENT 150
PATIENT 233
PATIENT 237
PATIENT 239
vanc
linz
dapt
32
16
8
4
2
1
0.5
0.25
no fcm
Daptomycin MIC(c) (µg/ml)
-100
0
100
200
Days from index sample
