# Supplementary material for: Daptomycin treatment impacts resistance in off-target populations of vancomycin-resistant Enterococcus faecium
Source: PLoS Biol. 2020 Dec 17;18(12):e3000987. doi: 10.1371/journal.pbio.3000987 (PMC7775125; doi:10.1371/journal.pbio.3000987)
Supplement: S1 Table — (PDF) [file pbio.3000987.s004.pdf]

S1 Table

Numbers of doses of daptomycin in the 6 months prior to the index samples and the number of days between the most recent dose and the index sample.

Patient_ID	Days since last dose	Doses last 6 months	Mean MIC_C
PID00004	0.32	12	2.38
PID00029	6.76	3	1.77
PID00032	0.86	4	3.10
PID00054	141.33	3	1.77
PID00064	60.93	11	2.59
PID00084	36.85	34	0.78
PID00086	56.77	6	5.03
PID00087	0.81	4	1.29
PID00150	0.06	12	7.43
PID00233	27.32	14	2.85
PID00237	51.90	11	1.77
PID00239	0.11	3	1.09
