# Supplementary material for: Daptomycin treatment impacts resistance in off-target populations of vancomycin-resistant Enterococcus faecium
Source: PLoS Biol. 2020 Dec 17;18(12):e3000987. doi: 10.1371/journal.pbio.3000987 (PMC7775125; doi:10.1371/journal.pbio.3000987)
Supplement: S1 Text — Distribution of Enterococcus species with patient samples and figures showing relationship between Shannon Diversity and mean or max MICCs and within-patient diversity. (PDF) [file pbio.3000987.s005.pdf]

S1 Text: Species Diversity

Enterococcal species diversity in the gut was obtained as a result of the sampling method used to isolate 20 *E. faecium* clones per sample, it is therefore worth noting that there is different sampling effort (for diversity) across samples. 14 out of 22 samples contained multiple species of *Enterococcus* with a further 3 samples containing a mix of VRE and VSE *E. faecium.* Visual examination of this diversity across samples shows no direct relationship to *E. faecium* daptomycin resistance (Fig 2).

We calculated Shannon Diversity score (H) for each sample using species level grouping. The species specific PCR identification allowed us to differentiate the four enterococcal species most commonly found in humans, *E. faecium*, *E. faecalis*, *E. casseliflavus* and *E. gallinarum*, all other clones were included as an Other *Enterococcus* group. Shannon Diversity showed no correlation to sample mean daptomycin MIC_C (Spearman $\rho = -0.06$, $p = 0.79$, Fig A(left)), or to the max daptomycin MIC_C from any sample (Spearman $\rho = -0.06$, $p = 0.80$, Fig A(right)). There was also no significant relationship between diversity and within-patient variation in MIC_C (mean SD of random effects) (Spearman $\rho = -0.36$, $p = 0.10$, Fig B).

Species diversity pre- and post- daptomycin exposure are only available for 3 patients. In Patient 4 both pre- and post- daptomycin only VR *E. faecium* were isolated. Patient 87 pre- and post-daptomycin populations are similar, both timepoints contained a mixture of VR *E. faecium* and other vancomycin resistant Enterococcus species. In Patient 150, pre-daptomycin there were no *E. faecium* isolated and post-daptomycin exposure only VR *E. faecium* was isolated. *The underlying data is available in S1 Data.*

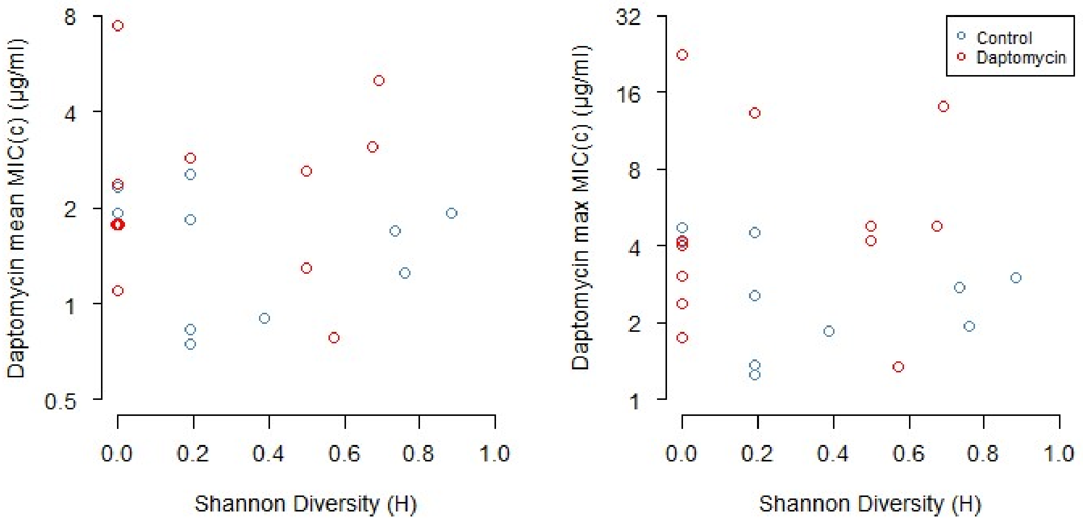

Figure A: Relationship between Shannon diversity (H) of Enterococcal species and mean MIC_C of clones isolated from an individual patient sample (left); or MIC_C of the most resistant clone isolated from each patient sample (right). Red circles are from daptomycin-exposed patients and blue circles are from control patients.

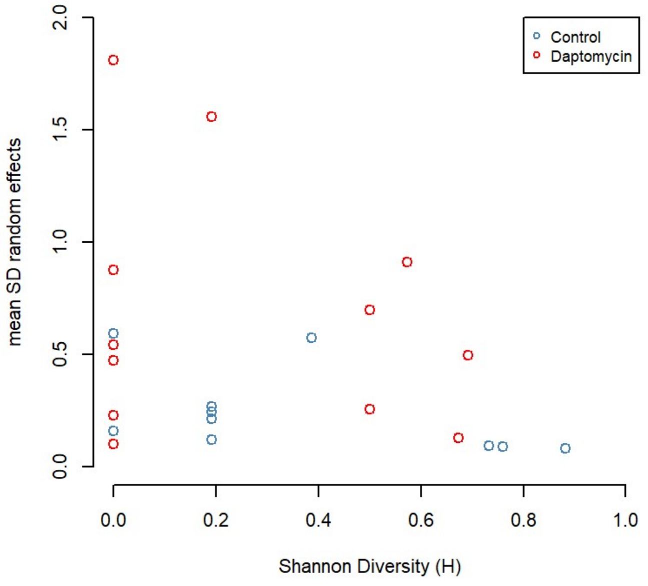

Figure B: Relationship between Shannon diversity (H) of Enterococcal species and within sample diversity in MIC_C (mean standard deviation of random effects – see Fig 4).
