# Supplementary material for: Daptomycin treatment impacts resistance in off-target populations of vancomycin-resistant Enterococcus faecium
Source: PLoS Biol. 2020 Dec 17;18(12):e3000987. doi: 10.1371/journal.pbio.3000987 (PMC7775125; doi:10.1371/journal.pbio.3000987)
Supplement: S2 Text — Full details of methods used to derive MICC values and full descriptions of models used for analysis. (PDF) [file pbio.3000987.s006.pdf]

S2 Text - Supplementary Methods and Analysis

Calculation of MIC_C – Fitting Dose Response Curves

Each plate has a column of blank readings. For each plate, we subtract the mean of this column from every reading on that plate. This adjusts for any plate specific noise and means that the expected OD reading of a bacteria free well is 0 (regardless of which plate it comes from). We denote this adjusted OD value by $OD_{adjusted}$. For each replicate of each clone we fit the following standard Hill function:

$$OD_{adjusted} = \frac{m}{1+(\frac{10^h}{x})^{-10^p}} \qquad (1)$$

where:

1. $OD_{adjusted}$ is the adjusted OD value, (the OD reading minus the estimate of the mean noise for the plate).
2. x is the drug concentration measured in $\frac{\mu g}{ml}$.
3. m is the maximum OD value (growth in the absence of drug).
4. 10^h is the drug concentration where growth is at half the maximum value (the OD50).
5. p determines the slope of the Hill function.

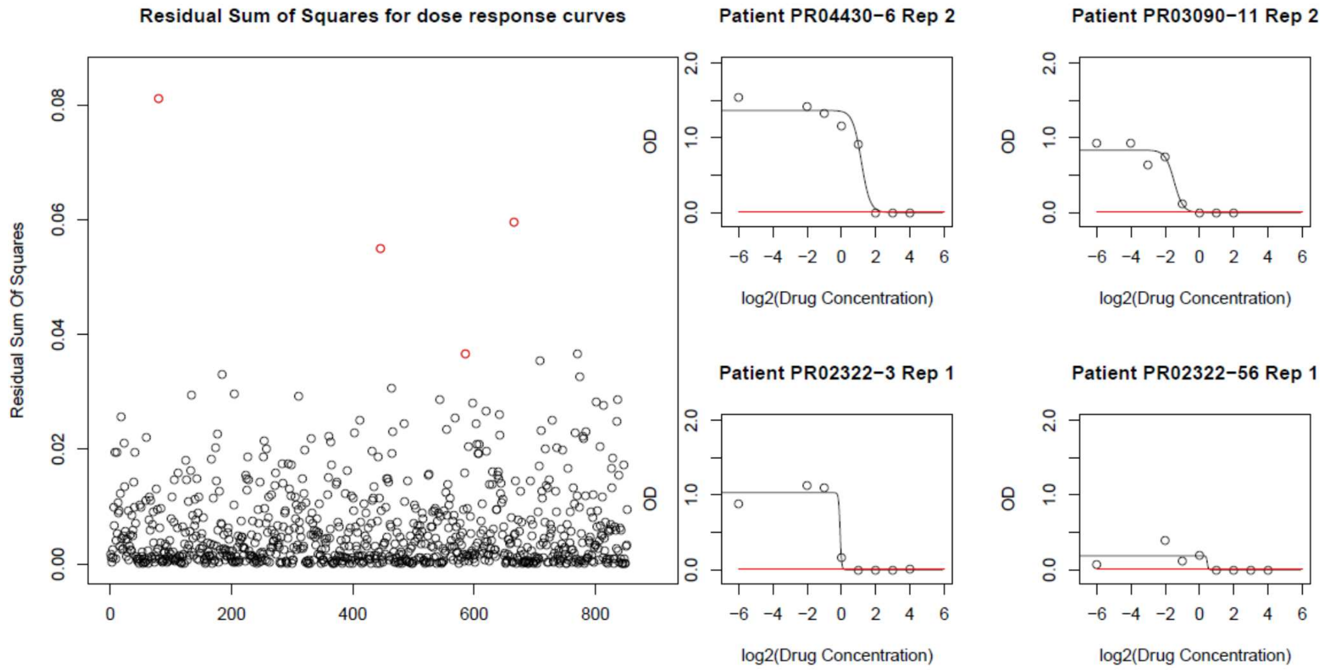

Fig A: Summary of fits for dose response curves. Left Panel: Residual sum of squares (RSS) for each fit. Red circles indicate the 4 highest RSS. Right Panel: Fits corresponding to the red circles in left panel. Clockwise from upper right: RSS=0.08112342, 0.05952475, 0.05498207 and 0.03654992.

For this study we have used MIC_C as our measure of resistance. This value is the closest continuous approximation of the MIC as it is the concentration where the Hill function predicts no growth of bacteria. See details of how the MIC_C is calculated below. This value is highly correlated with OD90 (Fig B).

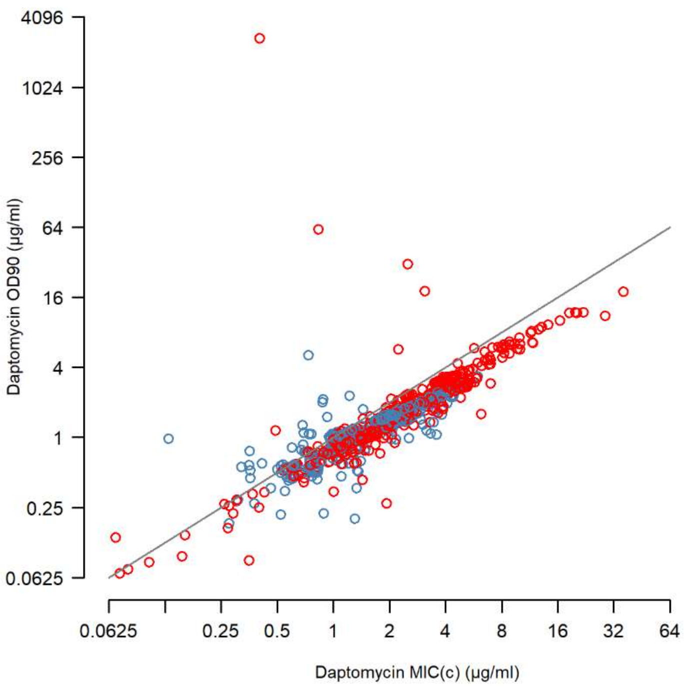

Fig B: Comparison of MIC_C and OD90. Each circle represents the OD90 and MIC_C of a single replicate. Red dots are for clones from the daptomycin-exposed group and blue dots are for control clones. The grey line indicates a 1:1 relationship between MIC_C and OD90.

Computing the computed MIC (MIC_C)

Using our fits to the data we use the following procedure to compute MIC_C values:

1. Using the procedure described above we have adjusted OD values for the column of blanks on each plate.
2. We find the mean ($Noise_{mean}$) and the standard deviation ($Noise_{sd}$) of all of these values.
3. We set the "detection threshold" to $Noise_{mean} + 2Noise_{sd}$.
4. We find the drug concentration were each dose-response curve intersects this detection threshold. We call this drug concentration the MIC_C.
5. Note that since we have two replicates per clone, this method will give us two estimates of MIC_C for each clone.

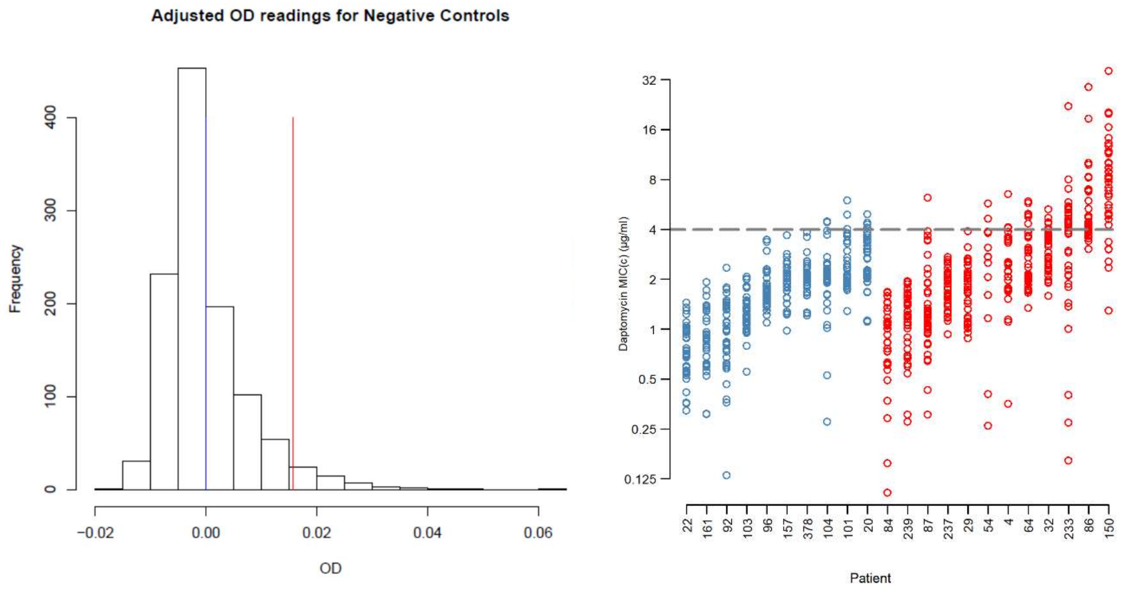

Fig C: Method for computing MIC_C. Left Panel: Histogram of all $OD_{adjusted}$ values for blank wells. This describes the distribution of the noise in the $OD_{adjusted}$ readings. The blue line indicates the mean and the red line the detection threshold. Right Panel: The computed MIC_C values for each replicate, using the method described above (blue: control patients, red: daptomycin treated patients). Black horizontal line indicates the cut-off for resistance in the hospital.

Statistical analysis of computed MIC (MIC_C)

The full model was

$$y_i = M_0 + M_D \chi_D(i) + M_{p[i]} + M_{c_p[i]} + \epsilon_i \qquad (2)$$

where

$$\chi_D(i) = \begin{cases} 1 & \text{if sample i comes from the dapto group} \\ 0 & \text{if sample i comes from the control group} \end{cases}. \qquad (3)$$

In Equation (2), y_i is the log base 2 of MIC_{C_i}, the MIC_C for the ith data sample (i.e., $y_i = log_2(MIC_{C_i})$). M_0 is the intercept and M_D is the fixed effect for belonging to the daptomycin "treatment" group. $M_{p[i]}$ is the random effect of "patient", $M_{c_p[i]}$ is the random effect of "clone" and ϵ_i is the random error associated with the ith sample. All random effects are assumed to be normally distributed with zero mean and a standard deviation whose posterior distribution is estimated via MCMC sampling.

In Equation (2) the distribution for the "clone" effect M_{c_p} can be different for each patient. We also considered different variations of Equation (2) by removing different random effects. Additionally, we considered the case where the random effect for "clone" was constrained to have the same distribution for all patients (in this case the term $M_{c_p[i]}$ is replaced by $M_{c[i]}$). Lastly, we considered the case where the clone effect depended on whether the patient was from the "treatment" group or the "control" group (in this case the term $M_{c_p[i]}$ is replaced by $M_{c_T[i]}$ where T=control or T=dapto). This final model (Model A) allowed us to directly assess if there was evidence for patients with prior daptomycin treatment exhibiting greater within-patient variability than the control patients. Specifically, $M_{c_T} \sim \mathcal{N}(0, \sigma_T^2)$ with $\sigma_T = \lambda$ for control patients $\sigma_T = \lambda \exp(\alpha)$ for treatment patients. With this model, if the posterior of α is predominantly above zero then this suggests there is strong evidence for the "clone" effect to be more variable for patients with a history of daptomycin treatment.

Table A lists the different models that were considered. Table B summarizes the possible combinations of random effects considered by the different models. The priors used in the analysis are given in Table B.

Table A: Model Summary

Model	
Model 1:	$y_i = M_0 + M_D \chi_D(i) + M_{p[i]} + M_{c_p[i]} + \epsilon_i$
Model 2:	$y_i = M_0 + M_D \chi_D(i) + M_{p[i]} + M_{c[i]} + \epsilon_i$
Model 3:	$y_i = M_0 + M_D \chi_D(i) + M_{p[i]} + \epsilon_i$
Model 4:	$y_i = M_0 + M_D \chi_D(i) + M_{c_p[i]} + \epsilon_i$
Model 5:	$y_i = M_0 + M_D \chi_D(i) + M_{c[i]} + \epsilon_i$
Model A:	$y_i = M_0 + M_D \chi_D(i) + M_{p[i]} + M_{c_T[i]} + \epsilon_i$

Table B: Model Summary

Prior distributions	dDescription
Fixed Effects	
$M_0 \sim U[-10,6]$	intercept
$M_D \sim U[-10,16]$	fixed effect for dapto patients
Patient random effect (used in Model 1, Model 2, Model 3 and Model A)	
$M_p \sim \mathcal{N}(0, \sigma_p^2)$	random effect due to patient
$\sigma_p \sim U[0,7]$	standard deviation for "patient" random effect
First version of clone random effect M_{c_p} (used in Model 1 and Model 4)	
$M_{c_p} \sim \mathcal{N}(0, \sigma_{c_p}^2)$	random effect due to clone
$\sigma_{c_p} \sim U[0,7]$	each patient has its own σ_{c_p}
Second version of clone random effect M_c (used in Model 2 and Model 5)	
$M_c \sim \mathcal{N}(0, \sigma_c^2)$	random effect due to clone
$\sigma_c \sim U[0,7]$	σ_c is the same for all patients
Third version of clone random effect M_{c_T} (used in Model A):	here T=control or T=dapto
$M_{c_{control}} \sim \mathcal{N}(0, \lambda^2\ )$	random effect due to clone for patients from control group
$M_{c_{dapto}} \sim \mathcal{N}(0, \lambda^2\ \exp(2\alpha)\ )$	random effect due to clone for patients from dapto group
$\lambda \sim U[0,7]$	λ is the same for all patients
$\alpha \sim \mathcal{N}(0,100)$	α is the same for all dapto patients
Random error:	
$\epsilon \sim \mathcal{N}(0,\sigma^2)$	random error
$\sigma \sim U[0,7]$	standard deviation of random error

The full model (Model 1) was determined to be the best model since it had the lowest DIC value (see main text for a summary of DIC values). General fit of the model was assessed by drawing from the posterior distributions of the selected model (Model 1) to simulate 1000 datasets and then comparing the actual data set to the simulated data sets. We considered three different statistics to assess how well the selected model characterized the actual data set. The first statistic was the mean of log2(MIC_C). Fig D shows the distribution of this statistic for all control patients (Panel A) and all daptomycin patients (Panel B). The bayesian p-values corresponding to this statistic are 0.485 and 0.491. The second statistic was the mean of the within-patient standard deviation of log2(MIC_C). P-values for this statistic were 0.122 for control patients (Fig E Panel A) and 0.229 for daptomycin patients (Fig E Panel B). The final statistic was the standard deviation of the within-patient standard deviation of log2(MIC_C). P-values for this statistic were 0.364 for control patients (Fig E Panel C) and 0.251 for daptomycin patients (Fig E Panel D). In all panels, the computed statistic of the actual data falls near the mean of the simulated distribution of the statistic. Although both statistics involving the within-patient standard deviation had slightly low p-values, overall the selected model seems to capture the main features of the data that were analyzed.

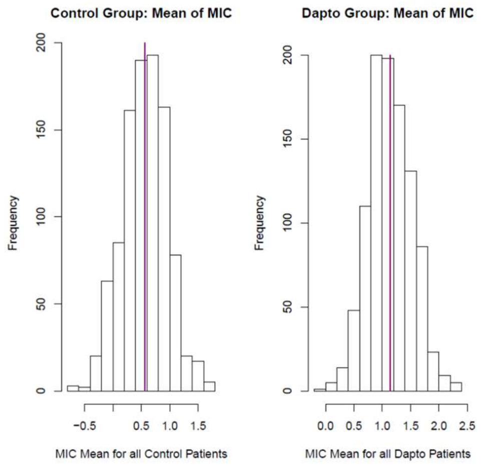

Fig D: Distribution of log2(MIC_C) for all control patients (Panel A) and all daptomycin patients (Panel B) for simulated data sets. Blue lines indicate means of distributions. Red lines indicate values for actual data set.

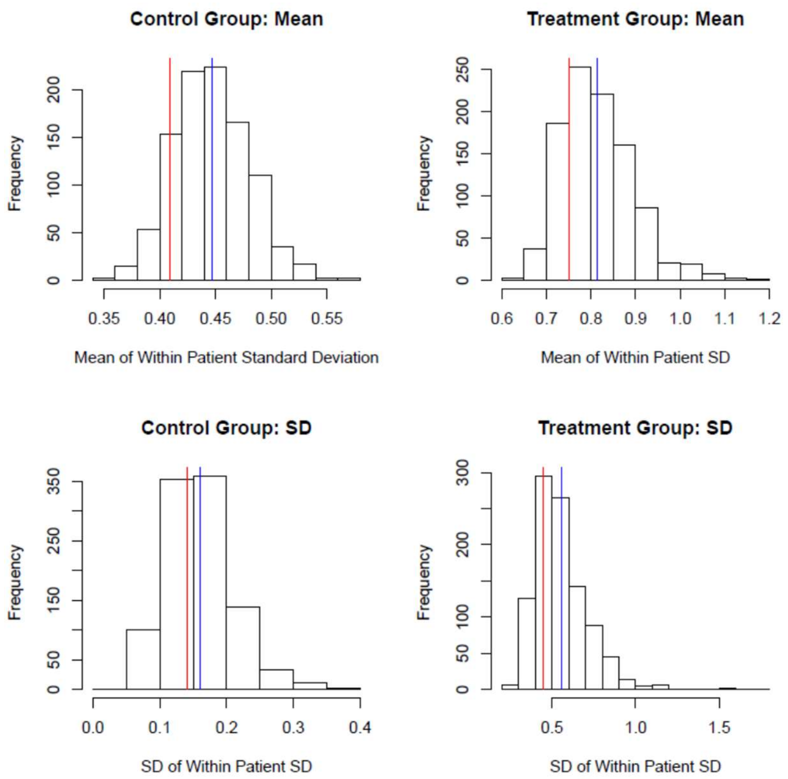

Fig E: Panels A and B show the distribution of the mean of the within-patient standard deviation of log2(MIC_C) for control patients (Panel A) and daptomycin patients (Panel B) for the simulated data sets. Panels C and D show the distribution of the standard deviation of the within-patient standard deviation of log2(MIC_C) for control patients (Panel C) and daptomycin patients (Panel D) for the simulated data sets. Blue lines indicate means of distributions. Red lines indicate values for actual data set.

Dataset Variations

A number of separate analyses were performed to assess how sensitive the results were to certain properties of the data. We considered three different manipulations of the data:

Variation 1: Removal of poor growing samples. There were a number of samples that grew poorly even in the absence of daptomycin. It's possible that the dose response of poor growers cannot be accurately characterized. To account for this possibility, we removed any data that was derived from a sample whose growth in the absence of daptomycin corresponded to an OD of less than or equal to 0.1. To see clones removed as low growers (Fig F).

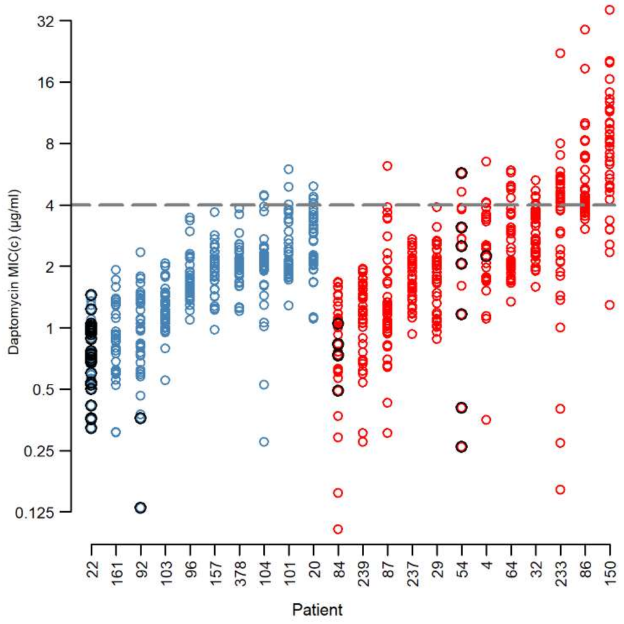

Fig F: Data removal for low growers MIC$_C$. Black points indicate samples that were removed from Variation 1 of the analysis. These points were removed because they were obtained from samples whose growth in the absence of drug had an OD of less than 0.1.

Variation 2: Removal of data from patient 8889. Patient 8889 was unusual compared to other patients in that it was difficult to isolate clones from this sample. Because of this, the final data set included data from only 8 clones of patient 8889.

Variation 3: Using OD90 instead of MIC$_C$. We also considered whether our conclusions about resistance depended on how we measured resistance. In addition to doing the analysis on the resistance measure MIC$_C$ we also did an analogous analysis on the OD90 (Fig G). The OD90 is the drug concentration where the dose response curve is reduced to 90% of its maximum and is well correlated with the MIC$_C$ (Fig B).

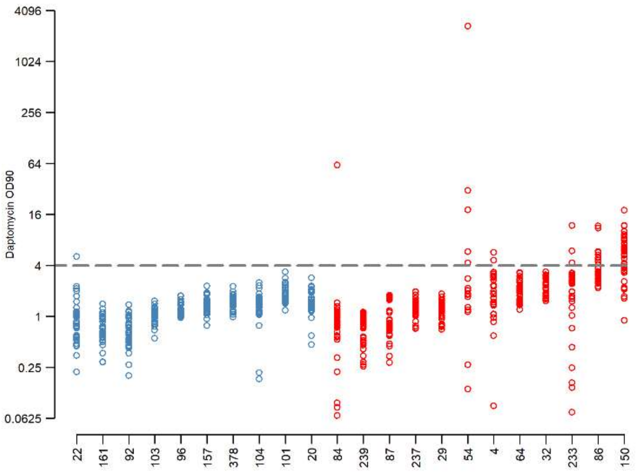

Figure G: Summary of OD90 as a resistance measure. log2(OD90) by patient. (Red: Dapto patients, Blue: control patients). Each data point represents a single replicate. The black horizontal line indicates the MIC cut-off for resistance in the laboratory.

For all variations, the model selected using DIC values did not change (see Tables C to E) indicating that the findings of the main paper are robust to these variations.

Table C: Variation 1 (remove low growers):
DICs for analysis of models listed in Table A

Model	mean deviance	DIC(pD)	ΔDIC
Model 1:	1034	1250	0
Model 2:	1065	1363	113
Model 3:	1699	1721	471
Model 4:	1051	1342	92
Model 5:	1066	1428	178
Model A:	1072	1316	66

Table D: Variation 2 (removing PR08889):

DICs for analysis of models listed in Table A

Model	mean deviance	DIC(pD)	ΔDIC
Model 1:	1095	1319	0
Model 2:	1127	1432	113
Model 3:	1774	1796	477
Model 4:	1109	1409	90
Model 5:	1127	1501	182
Model A:	1131	1396	77

Table E: Variation 3 (using $\log_2$(OD90)):

DICs for analysis of models listed in Table A

Model	mean deviance	DIC(pD)	ΔDIC
Model 1:	1236	1434	0
Model 2:	1421	1730	296
Model 3:	2052	2075	641
Model 4:	1321	1589	155
Model 5:	1399	1766	332
Model A:	1402	1619	185
