# Supplementary material for: Daptomycin treatment impacts resistance in off-target populations of vancomycin-resistant Enterococcus faecium
Source: PLoS Biol. 2020 Dec 17;18(12):e3000987. doi: 10.1371/journal.pbio.3000987 (PMC7775125; doi:10.1371/journal.pbio.3000987)
Supplement: S3 Text — Full lists of nonsynonymous mutations identified within patients and their locations on the phylogenies from Fig 7. (PDF) [file pbio.3000987.s007.pdf]

Within-Patient Evolution

Within-patient *E. faecium* populations were genetically diverse and evolved over time. Phylogenies for isolates from individual patients are shown in Figures A to D with data on daptomycin susceptibility for each isolate. Tables A to D list coding mutations detected among isolates within a patient. In several cases, variation was detected in genes previously associated with daptomycin susceptibility; however, in many cases, the evolution of increased daptomycin resistance occurred through previously undescribed genetic pathways. In addition to the variants listed in Tables A to D, many annotated genes were present in some genomes but not others. There were 215, 635, 280, and 2094 annotated genes present in some but not all isolates from Patient 4, 86, 87, and 150, respectively.

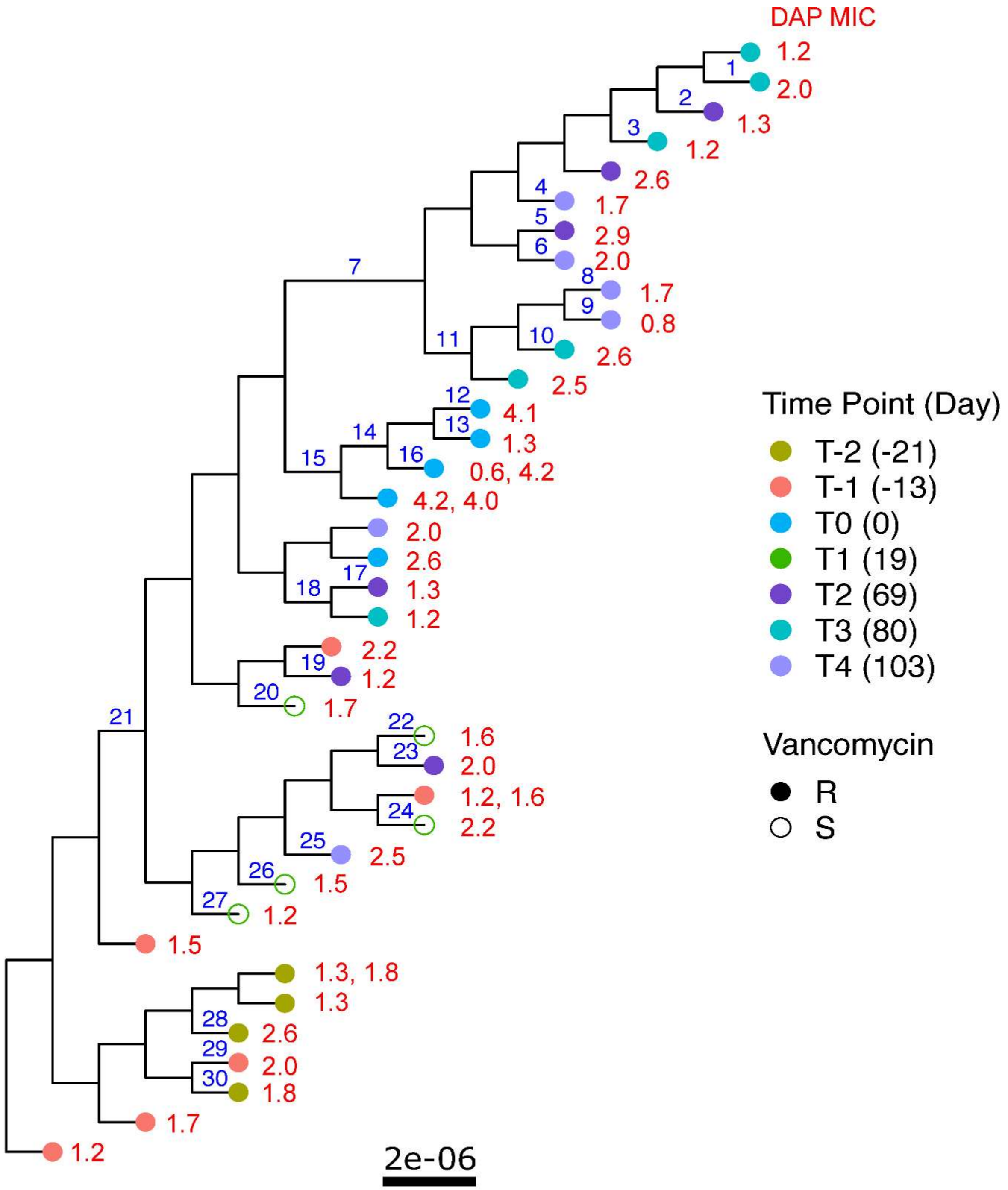

Figure A: Phylogeny for *E. faecium* strains isolated from Patient 4. Branch labels (blue) correspond to variants in Table A. For tips representing two isolates with identical core genomes, the daptomycin MICs for both isolates are listed (red). The index sample is labelled T0 and timepoints are labelled in order from that timepoint. The number of days between the index sample and the other timepoints are in brackets after the timepoint number.

Table A: Nonsynonymous mutations within Patient 4. Branch numbers correspond to Figure A. Asterisks indicates loci known to be associated with variation in daptomycin susceptibility [1].

Branch	Locus	Contig	Position	Mutation
1	hypothetical protein	1	2733759	S85F
1	*norG*	1	1200265	R87G
2	hypothetical protein	1	2142365	H140R
2	hypothetical protein	6	3320	I89ILG
3	*ctpE_2*	1	1780334	frameshift
4	5.5kb sequence	1	2636227	deleted: *yurK_1*, phosphorylated carbohydrates phosphatase, *manZ_5, levE*, hypothetical protein, *frlB_2*
5	5.2kb sequence	5	13746	deleted: 6 hypothetical proteins
6	hypothetical protein	4	40556	deleted
7	*fabD_2*	1	947147	Q162K
7	hypothetical protein	1	2324634	E264K
7	*murJ_1*	1	1395230	S144N
7	*rnpA*	1	2791463	E34D
7	*ybiT_1*	1	18596	TT359T
8	*potA*	1	689615	D167Y
9	*gmuR*	1	1026782	L211P
10	hypothetical protein	4	22620	18aa insertion
11	hypothetical protein	1	1966884	frameshift
12	hypothetical protein	1	1900362	frameshift
12	*pstB3_2*	1	1898781	frameshift
13	*rapA*	1	2355585	K488E
14	IS30 family transposase IS1062	1	1786098	frameshift
14	hypothetical protein	1	1900362	frameshift
15	*cls**	1	1036195	N13S
15	hypothetical protein	1	1521249	altered stop
15	ISL3 family transposase ISEfa5	2	199879	K429N
16	IS30 family transposase IS1062	1	1786098	frameshift
17	hypothetical protein	1	639610	frameshift
17	2.5kb sequence	1	930541	deleted: *prfC*, hypothetical protein
18	hypothetical protein	2	97613	deleted
18	hypothetical protein	2	99596	deleted
18	IS256 family transposase ISEfa13	2	106570	deleted
18	3.2kb sequence	2	101024	deleted: *panD, panC, panB,* IS256 family transposase ISEfa13, IS3 family transposase ISEfa8
19	*gshAB*	1	128400	E319K
19	hypothetical protein	1	1076147	Q259L
20	*rsmD*	1	1219512	G54D
20	*truA_1*	1	104779	A6V
20	*vanA* cassette	5	19816	deleted: *vanR, vanS, vanH, vanA, vanX*
21	*bceB_2*	1	744561	G352V
22	*vanA* plasmid	5	0	loss of *vanA*-containing plasmid
22	IS30 family transposase IS1062	1	1786098	frameshift
22	hypothetical protein	4	50960	frameshift
23	*comGA*	1	2389377	T148I
23	hypothetical protein	1	2539971	12aa insertion
23	*rihB*	1	1936471	S287N

23	*rplA*	1	2320420	P138A
24	*vanA* plasmid	5	0	loss of *vanA*-containing plasmid
25	*graS*	1	530930	Q110R
25	hypothetical protein	5	33751	deleted
25	*nylA*	1	1410475	F119S
25	*rex_2*	1	2101769	H102Q
26	1.5kb sequence	5	19818	deleted: *vanR, vanS*
27	1.5kb sequence	5	19818	deleted: *vanR, vanS*
28	hypothetical protein	1	1834542	frameshift
28	hypothetical protein	1	2558795	altered stop
29	hypothetical protein	4	50960	frameshift
30	IS3 family transposase ISPa31	2	115091	frameshift

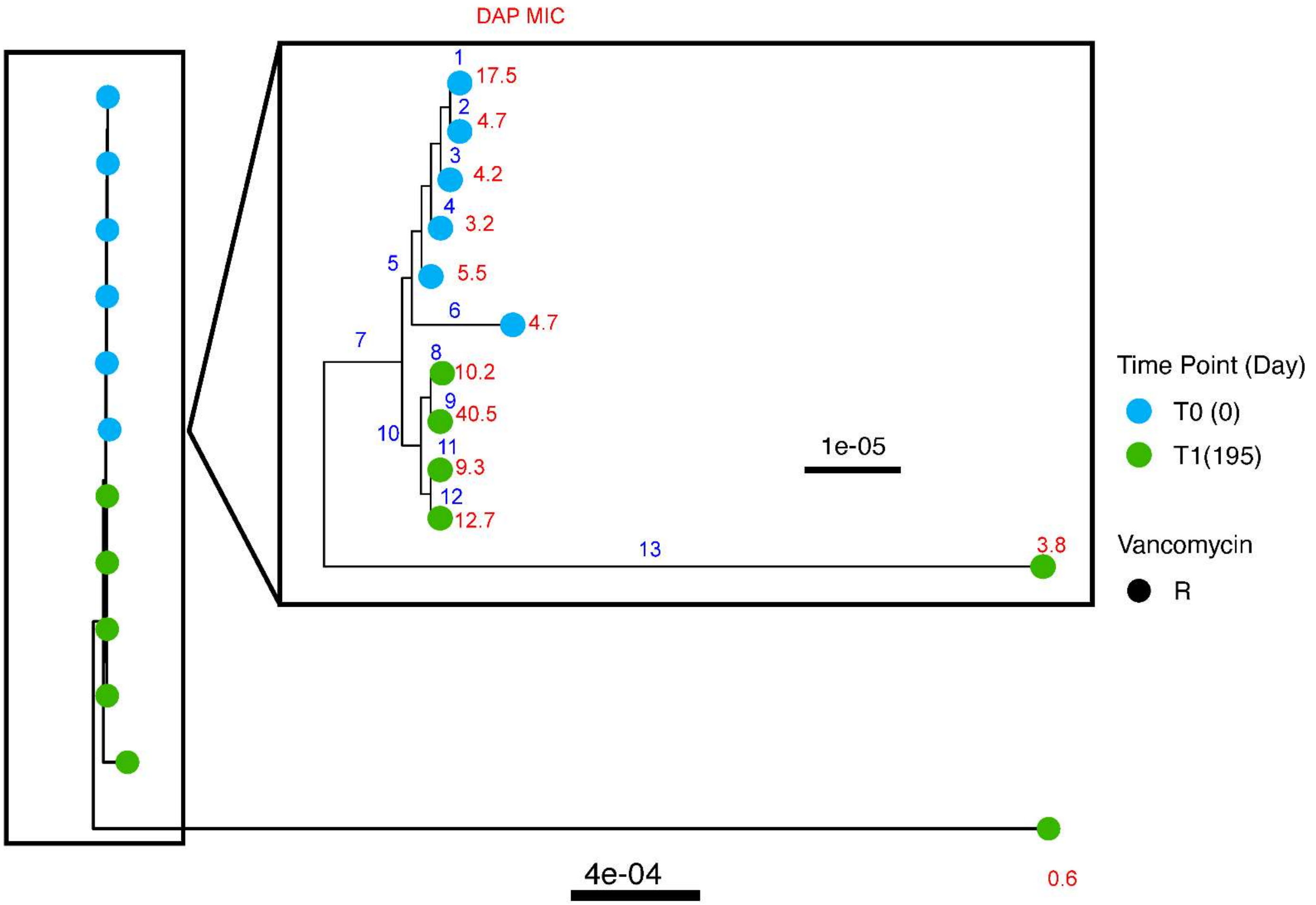

Figure B: Phylogeny for *E. faecium* strains isolated from Patient 86. Rectangle shows detailed structure of indicated group. Branch numbers (blue) correspond to mutations listed in Table B. The index sample is labelled T0 and timepoints are labelled in order from that timepoint. The number of days between the index sample and the other timepoints are in brackets after the timepoint number.

Table B: Nonsynonymous mutations within Patient 86. Branch numbers correspond to Figure B. Asterisks indicates loci known to be associated with variation in daptomycin susceptibility [1].

Branch	Locus	Contig	Position	Mutation
1	*kduD*	1	2112219	V128A
2	13kb sequence	2	70061	deleted: *panD, panC, panB*, multiple hypothetical proteins
2	208kb plasmid	3	0	plasmid lost
2	*gmuR*	1	960027	R14H
2	IS256 family transposase IS256, IS1595 family transposase ISCco2	9	3008	IS256 family transposase IS256, IS1595 family transposase ISCco2 deleted
3	208kb plasmid	3	0	plasmid lost
3	*MprF/FmtC**	1	936171	S455F
4	*pycA*	1	770546	D981E
5	50.1kb sequence	2	99381	deleted: large region of plasmid
5	*aspS*	1	1448994	G362E
5	hypothetical protein	1	1664819	P166Q
5	hypothetical protein	1	49743	frameshift
5	*YycG/Walk/VicK**	1	1375701	V356G
6	1.7kb sequence	2	56825	deleted: *licC_4*, IS1380 family transposase IS1678
6	2.4kb sequence	2	86579	deleted: IS3 family transposase ISPa31, *repN_1*, 2 hypothetical proteins
6	*citG_2*	2	130597	H233R
6	*dgaR_5*	1	2670675	V395G
6	hypothetical protein	2	74313	altered stop
6	hypothetical protein	2	104206, 104530	A18V, G126V
6	hypothetical protein	1	767470	hypothetical protein deleted
6	IS256 family transposase IS256	2	81564	F25V
6	IS3 family transposase IS1485	2	81564	R259K
7	*cls**	1	951390, 951820, 952557	F75V, R218Q, E464Q
7	hypothetical protein	1	821682	V227A
7	hypothetical protein	1	855641	D197G
7	hypothetical protein	1	974050	frameshift
7	hypothetical protein	1	2459968	A103E
7	*ohrB*	1	2371395, 2371612	A21E, L93F
7	*recQ_1*	1	776003	I289R
7	*scpA*	1	919169	S221G
7	*zosA*	1	755705	L245Q
8	50.1kb sequence	2	99381	reversion to plasmid variant with 50.1kb region
8	6-phosphogluconate dehydrogenase, NAD(+)-dependent, decarboxylating	1	490941	E220K
8	*citG_1*	1	2199727	frameshift
8	*ctpE_1*	1	109404	frameshift
8	*exuR_1*	1	855008	frameshift
8	*rpoC*	1	1807953	N675K
9	*exuR_1*	1	855009	altered stop
9	*lysA*	1	2335824	L253M

10	*rpsJ*	1	1948943	Y58S
10	*wecA*	1	18743	frameshift
11	*yadH*	1	1703267	E94D
12	42.5kb sequence	1	421827	42.5kb deletion in chromosomal genome
12	2kb sequence	1	566851	deleted: *cfa*, hypothetical protein
12	ISL3 family transposase ISEfa11	1	2825781	frameshift
12	*ygaZ_1*	1	586474	V136G
13	*agrA*	1	2311784	S32N
13	*asd*	1	2339953, 2340059	K176E, T211N
13	*bceA_1*	1	2292087	G221V
13	*bglF_1*	1	2219261, 2219983	I283T, S524P
13	*bglH_2*	1	2221306, 2221563	W334L, K420E
13	*cysL*	1	2428212, 2428277	L104P, T126A
13	*galM*	1	2351626	H76R
13	*garK*	1	2508631	A215D
13	*glpE*	1	2236403	L20I
13	*gpmA_2*	1	753085	T14P
13	hypothetical protein	1	794424	N137I
13	hypothetical protein	1	954110	D18E
13	hypothetical protein	1	1620289	12aa deletion
13	hypothetical protein	1	2288116	V81I
13	hypothetical protein	1	2304272, 2305000	E333G, T576P
13	hypothetical protein	1	2348001	frameshift
13	hypothetical protein	1	2361366	N228I
13	hypothetical protein	1	2426032	V99I
13	hypothetical protein	1	2435462, 2435480	A225T, E231K
13	hypothetical protein	1	2442088, 2443170, 2443264, 2443492, 2443522, 2443621, 2443654, 2443655, 2443682, 2443683, 2443685, 2443696, 2443698, 2443746, 2443751, 2443762	frameshift
13	hypothetical protein	1	2443920, 2443977, 2443979, 2444109	K26E, D69V, I70V, I89V
13	hypothetical protein	1	2444486	I58M
13	hypothetical protein	1	2444598, 2444850, 2444921	N12D, Q96K, MS119M
13	hypothetical protein	1	2445046, 2445057, 2445081, 2445082, 2445129, 2445158, 2445190, 2445222, 2445249, 2445270	R27K, S31T, V39I, V39E, T55S, D64E, T75M, V86I, V95I, T102A
13	hypothetical protein	1	2445481, 2445483, 2445513, 2445523, 2445531, 2445535, 2445543, 2445544, 2446053	A12V, I13F, F23L, Y26C, K29E, T30M, P33S, P33L, K203Q
13	hypothetical protein	1	2446957, 2447175, 2447183, 2447316, 2448041, 2448184, 2448271	altered stop
13	hypothetical protein	1	2448279, 2448313, 2448315, 2448905, 2449409, 2449509	frameshift
13	hypothetical protein	1	2450022, 2450463, 2450755, 2451093, 2451159, 2451252, 2451373, 2451463, 2451513, 2451622, 2451831, 2452056, 2452497, 2452499, 2452571	S167G, S314T, M411T, P524S, T546A, R577S, P617L, V647A, N664D, V700A, E770K, S845P, M992L, M992I, N1016K
13	hypothetical protein	1	2456963, 2457420	frameshift
13	hypothetical protein	1	2513899	L84F

13	hypothetical protein	1	2524098	H322R
13	hypothetical protein	1	2541370	H151Q
13	hypothetical protein	2	167232, 167450, 167456, 167675, 167757, 167884, 167987	A76S, V110A, E152D, E180K, Q253K, S255P, D327E
13	ISL3 family transposase ISEfa5	1	904571	K429N
13	*lacS*	1	2351881, 2352532	A264V, D481V
13	*lpdC*	1	2280438	T57A
13	*malH*	1	988842	R79C
13	*malP*	1	2262870	K731E
13	*metB*	1	2330242	frameshift
13	*metN*	1	2495987	D32G
13	*metQ_1*	1	2432982, 2433481	F8L, D175N
13	*mutS2_1*	1	2233684	frameshift
13	*npr_1*	1	2226194	E395K
13	*ogt*	1	2424863	R36Q
13	*pckA*	1	2364383	V206A
13	*pdhB*	1	784095	T101A
13	*pgaC*	1	2241447	Y78D
13	*pgmB*	1	2262578	T66S
13	*pheT*	1	725125	G372E
13	plasmid	9	0	plasmid lost
13	*purK*	1	810969	V82I
13	putative ABC transporter ATP-binding protein	1	2434196	D65N
13	*rhaS_3*	1	2354325, 2354382	K277E, T296A
13	*ribZ_2*	1	2367511	I176F
13	*rodA*	1	2429178, 2429808	L81F, I291V
13	*sufS_1*	1	2436202, 2436380, 2436607, 2436692, 2436773, 2436952	Y44H, T103S, F179I, M207R, V234A, K294E
13	*tdh_2*	1	2545115	altered stop
13	*topB_2*	1	2422422, 2422608, 2422631, 2423219	R347Q, G409E, A417T, L613M
13	*yfdH*	1	2424172, 2424178	I112V, C114S
13	*yjcD*	1	2317959	S528A
13	*yknY*	1	2459411, 2459412	altered stop

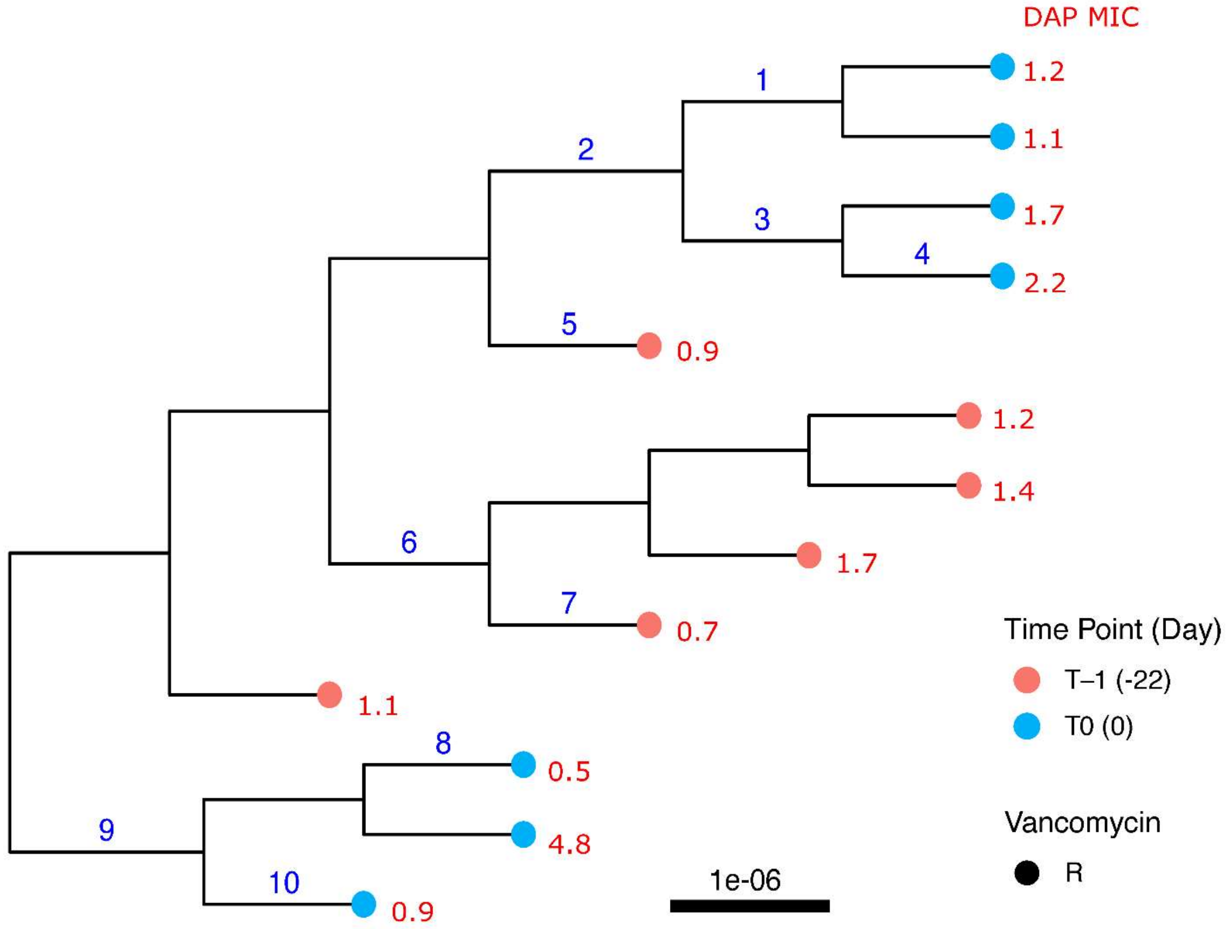

Figure C: Phylogeny for *E. faecium* strains isolated from Patient 87. Branch labels (blue) correspond to mutations listed in Table C. The index sample is labelled T0 and timepoints are labelled in order from that timepoint. The number of days between the index sample and the other timepoints are in brackets after the timepoint number.

Table C: Nonsynonymous mutations within Patient 87. Branch numbers correspond to Figure C. Asterisks indicates loci known to be associated with variation in daptomycin susceptibility [1].

Branch	Locus	Contig	Position	Mutation
1	*dgaR_2*	1	774236, 774239	frameshift
1	IS1182 family transposase ISBpu1	1	1853455	T43K
2	*cls**	1	1043463	R211Q
2	*rpoB*	1	2797760	G482D
3	*dgaR_2*	1	774309	L268Q
3	hypothetical protein	2	38574	I15M
4	2.1kb sequence	5	26621	deleted: 3 hypothetical proteins
5	3.2kb sequence	2	10397	deleted: 4 hypothetical proteins
5	4.8kb sequence	2	100691	deleted: *bin3_1*, 4 hypothetical proteins
5	hypothetical protein	5	33186	L617S
5	hypothetical protein	5	53745	W10L
5	*topB_5*	5	36699	Q488K
6	1.6kb sequence	5	8212	deletions: 5 hypothetical proteins
6	5kb sequence	6	2413	deleted: *erm*, IS1182 family transposase IS1182, *COQ5_3, aadK*, 3 hypothetical proteins
6	hypothetical protein	1	284855, 284911	frameshift
6	hypothetical protein	6	18062	I78N
6	hypothetical protein	6	24963	frameshift
6	IS30 family transposase IS1252	6	20420	D273E
6	plasmid	2	0	major structural changes in plasmid
7	*murI*	1	1276709	A258D
8	hypothetical protein	6	22634	deleted: hypothetical protein
9	*cls**	1	1043295	R267H
9	*rpoB*	1	2797749	H486Y
10	*cls**	1	1042998	P366L

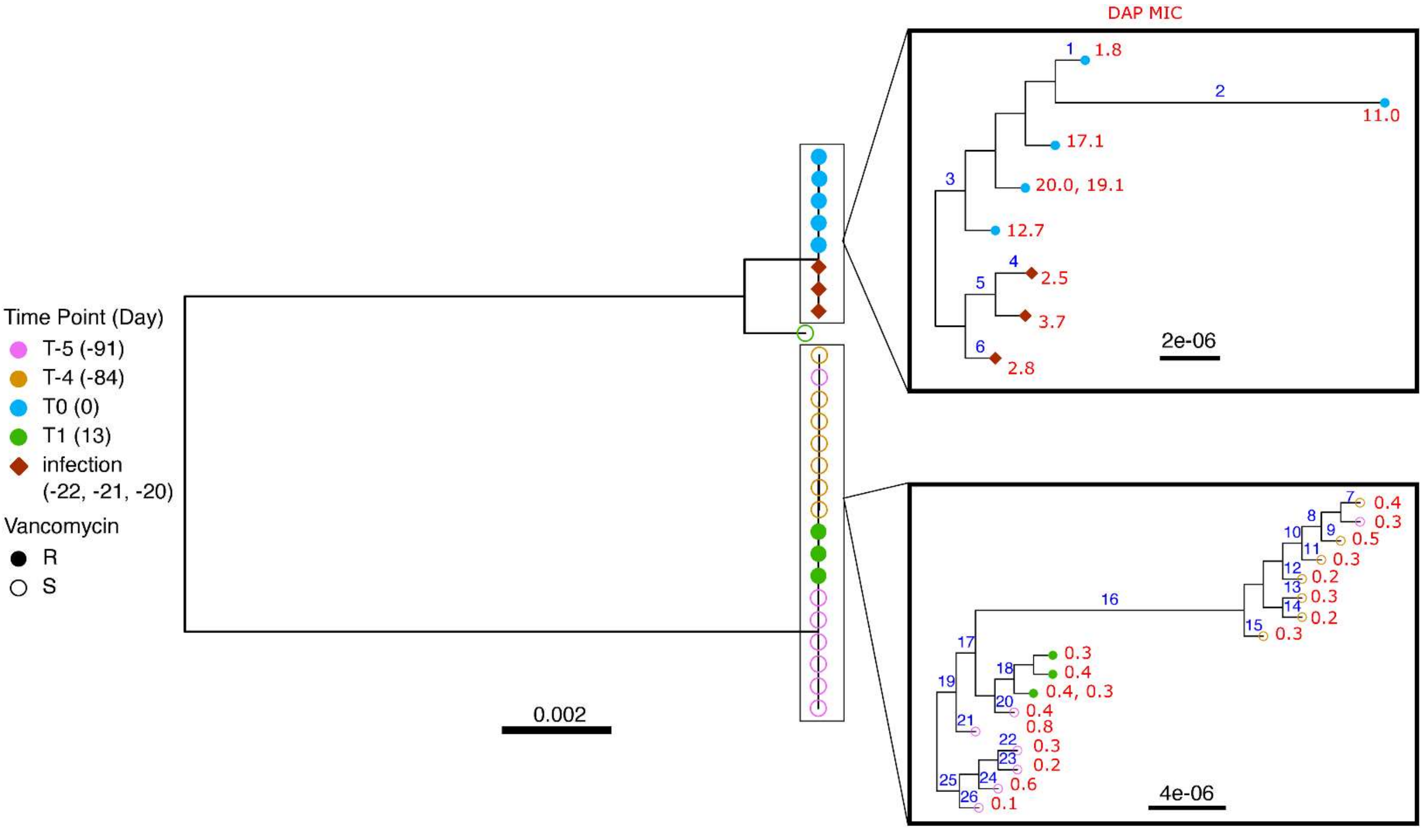

Figure D: Phylogeny for *E. faecium* strains isolated from Patient 150. Rectangle shows detailed structure of indicated group. Branch labels (blue) correspond to mutations listed in Table D. For tips representing two isolates with identical core genomes, the daptomycin MICs for both isolates are listed (red). The index sample is labelled T0 and timepoints are labelled in order from that timepoint. The number of days between the index sample and the other timepoints are in brackets after the timepoint number.

Table D: Nonsynonymous mutations within Patient 150. Branch numbers correspond to Figure D. Only variants for the upper box of Figure D are shown. The portion of the tree in the lower box included several apparent large recombinant regions and indels, which hindered assigning mutations to branches. Additionally, several large deletions were observed that are not listed in this table. Asterisks indicates loci known to be associated with variation in daptomycin susceptibility [1].

Branch	Locus	Contig	Position	Mutation
1	*murB*	1	1725616	P125S
2	7kb sequence	1	2549693	deleted: *ydcF, yhgF, pphA, tcyP*, SprT-like protein, 3 hypothetical proteins
3	7kb sequence	1	498760	inserted: 2 hypothetical proteins
3	41.5kb sequence	1	501602	inserted: *clpP_2, lexA_2, xerC_1*, 69 hypothetical proteins
3	*cls**	1	2030677	K61E
3	hypothetical protein	1	2519669	A32P
3	hypothetical protein	1	2529478	altered stop
3	35.8kb sequence	1	2717996	inserted: *xerC_9*, 57 hypothetical proteins
4	*ntpJ_2*	1	433972	G455C
4	*thrS*	1	1962103	R420H
5	*atpA*	1	150355	R279G
5	*gmuE_2*	1	1753158, 1753161	P259S, P260S
6	IMPDH	1	351508	V238I
6	IS30 family transposase IS1062	1	1333170	frameshift
7	hypothetical protein	1	2573572	S36L
8	hypothetical protein	2	113524	S68L
8	IS6 family transposase IS1216E	2	73619	altered stop
8	plasmid	2	0	plasmid loss
8	plasmid	5	0	plasmid loss with a few genes retained
8	plasmid	3	0	plasmid loss with a few genes retained
9	hypothetical protein	1	553389	T225A
9	IS3 family transposase IS1485	1	553833	F91S
9	*lipL*	1	723312	S93F
9	plasmid	4	0	plasmid loss with a few genes retained
10	hypothetical protein	3	25676	V145G
10	hypothetical protein	3	34483	frameshift
11	hypothetical protein	3	31374	frameshift
12	14.4kb sequence	3	22730	deleted: 13 hypothetical proteins
13	35.8kb sequence	1	1024245	deleted: *xerC_4*, 63 hypothetical proteins
13	*hdfR_1*	1	895114	R70K
13	hypothetical protein	1	1843110	V208I
13	hypothetical protein	3	30581	frameshift
14	hypothetical protein	3	30349	G454R
14	hypothetical protein	1	544446	frameshift
15	hypothetical protein	3	31374	frameshift
15	*rpoB*	1	2563777	I860V
16	17.1kb sequence	1	1238719	deleted: *pepT*, Putative lipid kinase, *mdtD, slyA, clpB, eis_1, lepA, cspLA_2*, 12 hypothetical proteins
16	hypothetical protein	3	25676	G145V
16	hypothetical protein	3	34483	frameshift

16	hypothetical protein	2	113524	L68S
16	IS6 family transposase IS1216E	2	73619	altered stop
16	*licC_4*	1	2397251	N298S
16	plasmid	5	0	plasmid acquired
16	plasmid	3	0	plasmid acquired
16	*rodA*	1	491860	M382V
17	hypothetical protein	1	219232	S107L
18	*vanA* plasmid			gain of *vanA*-containing plasmid, same plasmid that appears in upper part of tree
18	*glcR*	1	1170490	L4F
18	hypothetical protein	1	544446	frameshift
18	*lacE*	1	547652, 547828	T134M, F193L
18	*lacF_1*	1	548265, 548287, 548290	T86A, V87I, H94P
18	plasmid	2	0	variant/recombinant plasmid
19	*dprA*	1	1635002	altered stop
19	hypothetical protein	1	859495	altered stop
19	hypothetical protein	1	2519750	S101I
20	hypothetical protein	3	30902	frameshift
20	hypothetical protein	3	25676	G145V
20	hypothetical protein	3	34483	frameshift
20	hypothetical protein	2	113524	L68S
20	IS6 family transposase IS1216E	2	73619	altered stop
20	*pbpX_2*	1	1879598	T537A
20	plasmid	5	0	plasmid acquired
20	plasmid	3	0	plasmid acquired
21	plasmid	2	0	plasmid loss with a few genes retained
21	*ymdB*	1	125726	frameshift
22	hypothetical protein	1	544446	frameshift
23	hypothetical protein	1	1810322	frameshift
24	hypothetical protein	1	1810322	frameshift
25	hypothetical protein	1	133787	V83I
25	hypothetical protein	1	1279975	T15I
25	*pbpX_2*	1	1880201	G336R
25	plasmid	5	0	plasmid acquired
25	plasmid	3	0	plasmid acquired
26	*sasA_3*	1	1735414	L310S

References

1. Diaz L, Tran TT, Munita JM, Miller WR, Rincon S, Carvajal LP, et al. Whole-genome analyses of *Enterococcus faecium* isolates with diverse daptomycin MICs. Antimicrob Agents Chemother. 2014;58: 4527–4534. doi:10.1128/AAC.02686-14
