# Supplementary material for: Daptomycin treatment impacts resistance in off-target populations of vancomycin-resistant Enterococcus faecium
Source: PLoS Biol. 2020 Dec 17;18(12):e3000987. doi: 10.1371/journal.pbio.3000987 (PMC7775125; doi:10.1371/journal.pbio.3000987)
Supplement: Attachment — Submitted filename: Response to Reviewers.docx [file pbio.3000987.s009.docx]

We thank the editor and reviewers for their constructive comments for improving this manuscript. Please find below the original comments in black and our responses in blue. As was requested by the editor and two of the reviewers, we have now included sequencing and phylogenetics of a subset of our samples, we appreciate the request and feel that it has added significantly to our understanding of the dynamics in these populations. We think the manuscript is much improved as a result of the review process.

REVIEWERS' COMMENTS:


Reviewer #1:

The manuscript by Kinnear and colleagues examines how IV antibiotic use can drive selection of resistance in a bystander population of intestinal bacteria. The case control design and large numbers of clones tested from each patient are clear strengths of the study. Study limitations include a lack of connection between phenotypic and genotypic resistance, and ambiguity regarding some of the methods. The manuscript could be improved by addressing the following comments:

Major comments:

1. Phenotype-genotype connections. The authors report changes in daptomycin susceptibility phenotypes, but the lack of genotype data makes the paper seem very limited in scope. The authors went to great lengths to isolate numerous clones from their study patients, and selective genome sequencing would provide valuable insight into the population dynamics underlying the observed phenotypic changes. The patients described in lines 181-183 would be prime candidates for targeted whole-genome sequencing, and including this information would greatly increase the impact and relevance of this work.

*We appreciate the reviewer’s suggestion and have performed genome sequencing on the suggested patients (hence the addition of two authors). These new data are included in figures 6 and 7 and discussion of genotype-phenotype connections can be found from line 228.*

2. Focus on daptomycin and not linezolid. The authors use patients treated with linezolid as a control group, but presumably the same bystander selection could be happening in the linezolid-treated patients as well. Why not include information about how linezolid susceptibility changes as well, and have the two drug treatment groups serve as controls for one another? Do the authors believe that the evolutionary dynamics are different with linezolid versus daptomycin?

*Reviewer 1 is correct that bystander selection could also be occurring for linezolid. We did not test for linezolid resistance for two reasons: first, linezolid resistance is much less frequently observed among clinical isolates within our hospital (to date), and secondly, patients in the daptomycin cohort were not selected based on an absence of linezolid treatment, so a number of patients in the daptomycin group have also received linezolid. This can be seen in Supp S3. There is potential for the evolutionary dynamics to be different as the described linezolid resistance mutation are predominantly in de novo mutations of ribosomal proteins, or due to a small number of horizontally transmissible resistance elements.*

3. Lack of clarity in methods. This happens in two places: First, the paragraph describing isolation of clones (lines 101-114) is confusing, especially because it follows immediately after mention of screening for VRE. A topic sentence stating that VRE-positive rectal swab samples were used to isolate for multiple VR E. faecium clones from each patient would be helpful. Expanding Figure 1 to include the screening/isolation of clones from each patient would also increase clarity.

*We appreciate the reviewer’s suggestions to improve the clarity of the methods. We have added a topic sentence to line 108 (shown below) and have modified figure 1 include details of samples that were excluded due to isolation issues.*

‘Multiple *E. faecium* clones were isolated from each VRE-positive index sample using Enterococcosel agar.’

Second, use of the term "computed MIC" is likely to create confusion for readers accustomed to seeing MIC values follow a standard log2 scale. As described it sounds like the authors have calculated a value that more closely resembles an IC90, rather than an MIC. Unless there is literature precedent for the term "MICc" (which if so should be cited), using a different term to quantify drug susceptibility would help avoid confusion.

*We certainly appreciate the reviewer’s point of view and have added language to the methods to try to reduce confusion about our terminology. However, we feel that using a term similar to MIC is appropriate as the value we have calculated closely approximates the traditional visual MICs, as it estimates the minimum concentration that inhibits detectable growth. The continuous nature of the metric has the distinct advantage of allowing the statistical model fitting approaches need to test the hypotheses we’ve laid out. While the use of ODs and hill-functions are commonly used to calculate metrics such as IC90, this is different from our MIC_c_, as the IC90 measures proportional reductions in growth, rather than an absolute cutoff determined by a machines ability to detect growth or not. We have taken the opportunity to better describe the metric in line 413-419. If the reviewer or editor has an alternative term that they would prefer we would be happy to consider it.*

Minor comments:

1. The title is very broad and should probably be modified to include "Daptomycin" and "VRE."

*The title has been modified to read as follows:*

‘Daptomycin treatment impacts resistance in off-target populations of vancomycin-resistant *Enterococcus faecium*, a nosocomial bacterial pathogen.’

2. Consider including the concept of antibiotic tolerance in the Discussion. If daptomycin concentrations in the GI tract indeed reach >100ug/mL, then VRE with greater tolerance (which may or may not also have greater resistance) will be the ones to survive. For this reason, assays that quantify drug tolerance/killing might be more appropriate that an assay looking for overt "resistance" in this setting.

*We very much appreciate the potential role of antibiotic tolerance, but we avoided it in the discussion as we have not addressed this with data. We now acknowledge the possibility on line 337, and agree that is a very interesting direction for future work.*

3. The information on species diversity included in the S1 Appendix is worthy of inclusion in the main text, and its inclusion would increase the relevance of the study. Did species diversity change pre- vs. post-daptomycin exposure? Either result would be interesting, and should be discussed.

*We are pleased that the reviewer found this information interesting and have moved the figure S1-1A from S1 Appendix to the main text.*

*Species diversity data pre- and post- daptomycin exposure are only available for three patients so we added a brief discussion to the supplement S1. Due to the small number of patients where this data is available, we do not provide further analysis.*

4. Why were at least six doses of linezolid required for inclusion in the control group? Versus only three or more doses of daptomycin.

*Linezolid is dosed twice daily whereas daptomycin which is generally dosed once daily, therefore we chose 6 doses of linezolid as it reflects similar exposure to 3 doses of daptomycin. We have clarified this in the manuscript (lines 100-102).*


Reviewer #2:

Kinnear et al. investigate the extent of daptomycin resistance in enterococcal isolates from individuals with daptomycin exposure within the past 6 months and those without recent daptomycin exposure. They report that dapto exposure is associated with increased dapto resistance, that treated patients have a greater amount of within host diversity in the extent of resistance, and that the duration of colonization with dapto resistant VRE varies. Strengths of the study include its direct assessment of resistance in carried isolates and its comparison across multiple individuals recently dapto exposed / unexposed.

Although framed as an analysis of 'off-target' effects, the analysis is conceptually similar to defining the risk factors for resistance to a given antibiotic—and in that way, finding that exposure to daptomycin results in selection for colonization with daptomycin resistant organisms is in keeping with what has been observed for many other antibiotic-bacteria pairs. Similarly, the finding that duration of colonization with the resistant bacteria vary recapitulates what has been found in other analyses, including for VRE (<https://www.ncbi.nlm.nih.gov/pubmed/12002235>). It would be helpful if the authors would put their findings in the context of what's known about selection for other kinds of resistance in enterococcus and other GI-colonizing bacteria and how those isolates transmit. How do the authors' findings confirm, extend, and / or contrast with expectations based on the literature?

*We agree with Reviewer 2 that with data on exposure and a single sample after exposure it would be difficult to differentiate between selection for colonization by resistant bacteria and within-host selection for resistance. However, the genetic data requested by the other reviewers strongly supports our contention that this is off-target selection. From the phylogenetics of isolates from single patients over time (Figs 6 and 7) we can see persistence of genetically highly related isolates over time within patients with varying daptomycin resistance. This demonstrates that selection most likely occurs within the patient rather than by introduction of new resistance isolates after daptomycin exposure.*

*As mentioned in the introduction (lines 50-52), increases in resistance in gastrointestinal populations has been shown for a number of GI-colonizing bacteria following antimicrobial exposure. However, these studies typically look for presence/absence of resistance and are unable to determine the evolutionary mechanism for the increase. We have added a sentence to the introduction to highlight that this is lacking for previous studies (lines 55-58).*

The title refers to this as a case-control study. My epidemiological terms may well be rusty - could the authors clarify why this is a case-control, as opposed to a cohort study, given that the two groups were selected on the basis of different exposures?

*The reviewer is correct, because the groups were defined by the risk factor and not an outcome this is a cohort study. We have removed reference to case-control from the title and modified the methods to refer to it as a cohort study.*

A key part of the questions the authors set out to address has to do with resistance phenotype (as in the paragraph beginning line 69). The authors chose to generate a 'computed' MIC rather than stick with the standard MIC—could the authors please justify this choice? Given the expectation of error in MIC by broth microdilution testing for daptomycin in Enterococcus (Campeau et al., AAC 2018), how do the authors propose to allay concern that the analysis is overinterpreting the MICc and the variation they calculate, given the errors in measurement? Or do the authors think that the small (<~2 fold) MIC variations as plotted in Fig 2 are real and there are genetic/physiologic mechanisms that explain these variations (and if so, what support is there for this interpretation)?

*As noted above, we chose to use a computed MIC_C_ instead of a standard MIC as a resistance measurement for statistical convenience of a continuous metric, and the ability to automate the reading. We are very aware that testing has reproducibility issues (in fact, we have also studied this, Kinnear et all AAC 2019). While we agree that measuring daptomycin MICs is problematic, and Campeau 2018 show that there is variation in MIC due to media and between laboratories, in our hands we have found daptomycin testing to be repeatable (see Kinnear et al AAC 2019). Further, all the clones in this study have been tested with ≥2 biological replicates which allowed us to calculate the error term (*$\epsilon_{i}$*) in the model which, in fact, quantifies the measurement error between these replicates. This error is plotted in Fig 4, and we can see that for a number of patient samples (primarily in the daptomycin group) the within-patient variation (variation between clones within a patient) is significantly greater than the between-replicate (testing) variation.*

In that paragraph, the authors also articulate a foundational hypothesis for the study, but it's a bit confusing. Could the authors clarify what they mean by "most transmitted" daptomycin resistance is due to off-target selection? It seems like this manuscript doesn't investigate transmission, but instead focuses on within-host dynamics of daptomycin resistance. Also - what's the alternative hypothesis, and wouldn't many of the questions pertain even if the hypothesis is false (even a small amount of transmitted resistance comes from the gut)?

*We have modified the wording to remove the term ‘transmitted’ daptomycin resistance (line 76) as it was raised by multiple reviewers. Our point was: as transmission of* Enterococcus *is by the fecal-oral route, it is more plausible that off target selection is responsible for the generation of resistant isolates that are transmitted within the hospital than on-target selection and subsequent transmission of isolates from sterile site infections. This study looks at the first step of this, which is that antibiotic exposure results in increase resistance in the off-target population.*

Risk factors for VRE colonization include hospitalization and antibiotic use (as well demonstrated in Fig 4, where the patients have multiple hospitalizations over the course of months). How does use of other antibiotics (e.g., cephalosporins, a known risk factor for VRE) impact their observations? Could there be selection for the dapto resistant strains through co-resistance? (e.g., to quinolones or other antibitoics?)

*A recent large retrospective case-control study within our hospital (Chanderraj et al OFID 2019) showed that, other than vancomycin, all antibiotics confer similar increased risk for VRE acquisition. There is no clear clinical data to indicate cross resistance between daptomycin and other antibiotics in enterococcus. In laboratory settings, the collateral resistance in Enterococci that has been described is inconsistent and weak (Bhardwaj et al AAC 2017, Maltas and Wood Plos Biology 2019), thus we had no prior predictions about antibiotics to look for collateral sensitivity/resistance. We have included the limitations of a retrospective cohort study design in the discussion (line 338-339).*

Were the patients in the Time Series section drawn from those in the rest of the study? If so, could you identify in Figure 4 which samples contributed to the other sections of the study?

*Yes, the patients in the time series are a subset of patients from the main study where we had access to multiple samples over time. The main section sample is always the 0 timepoint for each patient. We have added the following sentence to the figure 4 (now figure 5) caption to clarify this.*

‘Time 0 is the index sample included in the previous analysis.’

Why was 6 months the limit for evaluating prior exposure? If you look back further, were there other exposures in either cohort?

*While the exact turnover of VRE in the microbiome is unknown, 6 months to a year have been suggested as averages. We chose to limit antibiotic exposuse to 6 months for inclusion in the treatment and control cohorts to reduce the chance that patients with distant drug exposure would dilute the effect of daptomycin exposure even if it exists.*

*However, we included all available drug data when determining patient cohorts. For the control patients, there was no daptomycin treatment on record in the electronic medical record database which for these patients has records as early as July 2011. We have changed line 362 to state that control group patients had no known daptomycin exposure.*

Minor comments

Line 30. Suggest reporting "50% higher" in terms of MIC dilutions

*Conversion to log_2_ units, or 2 -fold dilutions, is immediately interpretable to a portion of the audience (clinical microbiologist and clinicians) for this paper, we opted for 50% higher as a more generally interpretable expression of the results for the PLOS Biology audience, which we believe is more broadly include scientists and policy makers. We are open to editorial recommendations on this point.*

*One doubling is 100% higher, so our finding of 50% higher is half that, or half of a 2-fold dilution.*

Lines 70-72. I'd guess there are studies that look for risk factors for daptomycin-resistant VRE, including past daptomycin use (e. g., <https://www.ncbi.nlm.nih.gov/pmc/articles/PMC6176497/>). Are the authors suggesting that evaluation of resistance has only been identified in the population causing an infection, and that population then persists in some way after treatment for infection and seeds future infections?

*There are a number of studies that look for risk factors for daptomycin-resistant VRE, including past daptomycin use, as we mentioned in the prior paragraph, however these look at infections. We are not suggesting that the infection population persists after treatment, but that the dynamics in the infecting population may be quite different from those in the colonizing population.*

Line 110-3. Construction is confusing: how is 9/22 isolates with a combination of VRE and VSE a majority?

*We were trying to make the point that the majority of sample were not VR E. faecium only, rather they contained either multiple species or mixed VRE/VSE. To make this clearer we have modified the sentence to read:*

‘The majority of patient samples (17/22) were not homogeneous, with 14/22 containing multiple enterococcal species and 9/22 containing a combination of vancomycin resistant and vancomycin sensitive *E. faecium*.’

Methods - what if anything was done with the isolates from blood stream infection?

*Resistance of the blood stream isolates are in S3 Appendix: Time Series Plots for All Patients. Also, for Patient 150, three blood stream infection isolates have now been sequenced.*

Suggest clarifying in Figure 1 the 3 days of therapy. Why was 3 days chosen as the lower limit?

*Three doses of daptomycin (usually equal to three days of therapy) was selected based on our understanding of common treatment timeframes for enterococcal blood stream infections where it is rare for patients to receive less than three days of therapy (see Kinnear et al AAC 2019). We have added the following to the methods (line 360) to clarify this:*

*‘Three doses was selected as a minimum requirement because treatment of enterococcal infections generally requires at least three days of treatment’*

Fig 2 legend. What do the authors mean by 'equivalent to the clinical 2-fold MIC cutoff'? I think the authors are trying to clarify that, given that MICs are only determined in 2 fold dilutions, an MIC of >4 will appear as ≥8 - is that correct? However, it seems they might be suggesting that an MIC of 4 is interpreted as an MIC of 8 in clinical settings.

*Yes, the former is correct. For clarity we have modified the Fig 2 (now Fig 3) legend to read:*

*‘due to the continuous nature of the computed MIC (MIC_C_), an MIC_C_ of >4μg/ml is equivalent to the clinical 2-fold MIC cutoff of ≥8μg/ml’*

Fig 4. What is "fcm"? Please explain all abbreviations in the Fig 4 legend. Patient 150 seems to have a bunch of VSE without any VRE at ~-100. How were these obtained, if inclusion in the study required VRE?

*Fcm referred to E. faecium. We have modified the Fig 4 (now Fig 5) legend to contain the full species name. The inclusion criterion was that the index sample (Time 0 on the timeseries) screen positive for VR E. faecium. For the timeseries analysis, all samples available from the patients identified for the main study were sampled for any E. faecium. We have added ‘E. faecium’ to line 193 to clarify that this is all E. faecium and not just VRE.*

Fig 2 and Supplemental Fig S1b look identical.

*This is correct, we thought it was useful to have the direct visual comparison in the supplementary material. As reviewer 1 has requested that this supplementary material be included in the main text, this figure will no longer be duplicated.*

Fig S2-2. Please label the MICs, rather than just using relative size

*We have added MIC labels to Fig S2-2.*

Reviewer #3:

The submission by Kinnear et al. examines the important question of how antibiotic treatment using daptomycin on E. faecium may select for increased daptomycin resistance within the E. faecium population of the gut essentially laying the foundation for new daptomycin resistant strains as an unintended consequence.  From a collection of rectal swabs within a single hospital site 6,726 patients were screened for vancomycin resistant enterococci (VRE). Of these, 618 were VRE positive. It is not clear why only VRE strains were of interest (though the clinical relevance is clear). Off target effects could have been more broadly survey initially from the broader enterococci and then the authors could drill down on the VRE strains to see if there was something special genetically (and thus phenotypically) that would be characteristic in pre-disposing their ability to become resistant to daptomycin.  From this group 23 had been treated with daptomycin and made up the initial study group with another group treated with linezolid used as a control (n=15).  Strains were isolated and the MIC to daptomycin determined using standard CLSI methodology. As expected the patient group that had been treated previously with daptomycin held a substantially more diverse population of daptomycin resistant phenotypes presumably as a result of off-site selection.

*We agree with the reviewer that sampling Enterococcus more broadly would be interesting and relevant. The decision to focus on VRE positive samples was a practical one, as among the >6,000 samples, the VRE faecium positive samples had already been identified. The process of re-screening for VSE faecium is more labor intensive. Thus, it would have been unfeasibly time consuming to initially screen for all E. faecium. Further, including other species of Enterococcus for MIC testing would have reduced our ability to test for changes in resistance rather than just changes in species (which often have inherent differences in resistance) composition.*

 Of interest is the fact that daptomycin is administered by IV and not orally so the compartmentalization of the drug might have suggested that off site deletion would be less. Clearly, orally administered antibiotics get into the gut and there have been studies showing off site selection in that more expected context.  The authors cite a previous work that 5% of the administered daptomycin can be observed in the feces of patients but I found the estimate of 125 to 250 micrograms/ml extraordinarily high seeing as the clinical breakpoints are in single digits. Nearly all Gram positives would be eliminated by such extremely high doses. Ref. 37 is about a study where C14-daptomycin was administered to patients and recovery of C14 in urine and feces was tested. Fig. 2 in that paper states that cumulative C14 recovered from feces was 5% but it does not mean that the recovered C14 was intact daptomycin. In fact, the same figure shows that C14 recovery in urine was 78% and that corresponded to 57% daptomycin.

*We agree with Reviewer 3 that there remains significant uncertainty about the metabolism and excretion of daptomycin. It is highly likely that some of the daptomycin is inactive at this sight and we also assume that there is likely niche variation within the gut that provides a variety of selective environments. Our goal was simply to review the limited available data. We have added a comment to the discussion to note that the active concentration is likely lower than the total concentration. Clearly this is an issue and exactly part of the point of this paper. These off-target effects are rarely considered and very little data exists despite the considerable impact that they are likely having on resistance.*

 Unfortunately there are quite a few gaps in the study. Sequencing and phylogenetics of isolated strains are to be expected in this day and age and none is performed in this work.

*We have now sequenced multiple clones from each timepoint from Patients 4, 86, 87 and 150 (Fig 6 and 7) and included a phylogenetic analysis.*

Many wonderful studies have been performed with single and systems of hospitals tracking the movement of strains such as A. baumannii from beds to workers etc. This paper merely shows that patient treated with daptomycin are potential reservoirs for reinfection or colonization of the hospital across patients/locations. This does not surprise the reviewer as it has been shown in many other cases.

*We agree with the reviewer that there are many good studies of transmission of drug resistant pathogens within hospitals. However, to our knowledge there are no studies directly showing that increased resistance in hospitals can be driven by off-target selection.*

The author speculate (lines 69-75) that off target selection and the associated evolutionary dynamics would be important to know but they have the collection of strains and expertise to determine and test that hypothesis.

*We completely agree! The long-term project of our group is to understand both the transmission dynamics and evolutionary dynamics of this* E. faecium *in the hospital setting, and measure their impact on human health. We think that the work presented here provides a key step forward by identify the role of antibiotic exposure in resistance emergence, the compartment in which selection is happening, the impact of drug exposure on within patient phenotypic diversity, and with the genetic data that has been added, preliminary molecular data. There is indeed much more work to be done on this system.*

General comments on the paper:

1.      What is the normal level of diversity in daptomycin resistance in the gut flora? The test group in this study included patients treated with daptomycin and the control group had patients treated with linezolid. The linezolid in the control group could have cleared some of the daptomycin resistant members of the gut in the control population. Another control group of individuals not treated with antibiotics would have been more informative. (The gut bacteria are exposed the host derived cationic antimicrobial peptides that are similar in mechanism to daptomycin. This exposure can also induce resistance and thus, a control patient population not treated with any antibiotics will be a better baseline for this study).

*We understand the reviewers concern in the choice of control group, and impact of antibiotics. We chose this group to try to select a similar group of patients (as daptomycin and linezolid are used to treat similar conditions) as a control. We note however that the selection criteria for the daptomycin group did not include the absence of previous linezolid treatment and 7/12 patients in this group had previous linezolid exposure which can be seen in Supplementary Material 3. Two of the three patients with highly daptomycin resistant clones had prior linezolid exposure so we feel that it is unlikely that the lower resistance in the control group is driven by linezolid exposure.*

*We note that our previous study of VRE acquisition in this same hospital (Chanderraj et al, OFID 2019), found that ~97% of patient with VRE positive swabs had received antibiotics. Informal review of patient who did not receive antibiotics showed that they were not typical of the VRE colonized patients (i.e. generally older, with more medical co-morbidities), so we felt use of this group as a control would have been less informative, or at the very least, raise questions about a host of other confounders that would be difficult to evaluate.*

2.      It is surprising that E. faecium isolates from only 3 out of the 12 daptomycin treated patients showed high levels of daptomycin resistance. A very small number of clones from other patients crossed the MIC threshold of 4µg/ml and even so, they did not differ very much from the control patients 104, 101 and 20.

*While it is true that we only see highly resistant isolates in 3 out of 12 patients, it is not entirely unexpected given prior knowledge of this system. Daptomycin resistance seems to be can be somewhat constrained within patients, with daptomycin MICs from blood stream infections rarely being higher than 8µg/ml (Kinnear et al, AAC 2019). Further, the selective pressure imposed by the daptomycin was variable within the group with 6/12 patients in the treatment group having less than 5 days of treatment and 6/12 patients having their last dose of daptomycin ≥4 weeks prior to the sample.*

3.      Lines 202-211: Among the 12 patients tested, data was only available from 3 patient samples to determine if daptomycin resistance was maintained after cessation of treatment. Within this small sample size, clones from 2 of the 3 patients showed rapid loss of daptomycin resistance. Did the authors measure the fitness cost of acquiring daptomycin resistance? This could provide some insight into the reason for the rapid loss of resistance.

*We agree that this rapid loss, and variable trajectories of resistance are very interesting. We have not measured fitness costs of daptomycin resistance because fitness depends upon the environment and the competitors. It was not clear to us that relative fitness in laboratory conditions, absent of the complex biotic and abiotic condition of the gut, would be relevant. As we noted in the manuscript, the rapid loss of resistance in these two patients also coincided with the presence of VS E. faecium in the latter sample that was not present in the index sample, thus isolating to the fitness cost due to daptomycin resistance alone, would not be trivial, and would ideally include isogenic strains with daptomycin resistance mutations we have now identified with sequencing results. Reverse genetics with E. faecium remain a challenging endeavor.*

4.      In line 69, the authors hypothesize that off-target selection in the GI tract is the cause for most transmitted daptomycin resistance in VRE. However, this work shows no association between the off-target daptomycin resistance in fecal samples and the transmission of daptomycin resistance. Yet, in line 224 it is stated that this could be a plausible cause of evolution of daptomycin resistance in hospital. This statement seems a bit far-fetched, especially when resistance was rapidly lost in 2 out of 3 patients whose long-term samples were available.

*Daptomycin resistance did increase in this hospital, including among patients without prior daptomycin exposure (Kinnear et al, AAC 2019). There are numerous potential explanations for this. While 2 of the 3 patients had reversion, 1 showed remarkable maintenance of resistance, even in the abscess of antibiotic pressure. We continue the believe is very plausible that a subset of daptomycin resistance the evolves in the off-target population contribute to the observed hospital wide resistance trends. The alternative is that transmission of bacteria from sterile site infections is the major cause of hospital transmission, and while possible, it seems less likely with best practice than fecal transmission. The reviewer is correct that we didn’t directly measure this, which is why we qualified the statement with the word “plausible”.*
