# Supplementary material for: Daptomycin treatment impacts resistance in off-target populations of vancomycin-resistant Enterococcus faecium
Source: PLoS Biol. 2020 Dec 17;18(12):e3000987. doi: 10.1371/journal.pbio.3000987 (PMC7775125; doi:10.1371/journal.pbio.3000987)
Supplement: Attachment — Submitted filename: Response to Reviewers.docx [file pbio.3000987.s010.docx]

REVIEWERS' COMMENTS:


Reviewer #1:

The manuscript is much improved from the initial submission, and the inclusion of the genome sequence data greatly increase the importance of the work.

*We thank Reviewer 1 for their initial review and are pleased that they believe that the manuscript has improved both in content and importance with the inclusion of the genomic work.*

I only have a few minor comments:

1. Consider abbreviating "computed MIC" as cMIC, rather than MICc.

*We thank the reviewer for the suggestion, however another paper from our research group was accepted for publication this week using the MIC_C_ abbreviation, so we would prefer to leave it as is for consistency. We are also slightly concerned that the prefix ‘c’ often means circa (about). However, happy to follow editorial advice here.*

2. Line 71: "VRE" is not defined before this use. Consider changing to VR E. faecium.

*We have made this change.*

3. Figure 6 - is the MIC axis title meant to be MICc/cMIC?

*Yes, we have updated this axis title.*

4. Figure 7 - are the blue asterisks primarily meant to show clsA mutations? I suggest adding a blue asterisk to the figure key, and also listing the actual mutations themselves on the phylogenies. Also in the text Fig. 7D is discussed before Fig. 7C - either the figure panels should be flipped or the text should be re-organized. Finally, a simple schematic of the bacterial population dynamics over time in Patient 150 as revealed by WGS would be helpful.

*Thank you for these suggestions. We have swapped panels C and D on the figure so that they are discussed in order.* *The blue asterisks designate mutations that have been demonstrated to effect daptomycin resistance. These are mainly cls but also MprF/FmtC and YycG/Walk/VicK. We have changed the color of the asterisk to reflect the specific mutation and have added these in the image legend.*

*Based on the suggestion from the reviewer and feedback from the editor we have added a supplementary figure (S3 Fig) with a schematic for Patient 150.*

Reviewer #2:

The authors have substantially revised their manuscript, adding genome sequencing and phylogenetic data. They expand on their main point -- that use of daptomycin selects for increased resistance in gut-resident enterococci ("off target" effect), which are transmissible -- with comments on the dynamics and variability of phenotypic resistance and genotypic evidence in support of within-host evolution of resistance.

I appreciate the authors addressing many of the items raised in the first round of review. For my point about resistance to other antibiotics, it wasn't that I expect daptomycin resistance to confer resistance to other antibiotics; instead, I meant co-resistance: isolates that develop dapto resistance may be selected for if they are also resistant to, say, quinolones, and quinolones are also used on the patients. To the extent that there is variation in susceptibility to other drugs and use of those drugs in patients, this may explain variations in persistence of daptomycin resistant lineages.

*We appreciate the reviewer clarifying this comment. We agree that many factors other than daptomycin susceptibility may contribute to the variations in persistence of resistance and that exposure to other drugs is likely to be one of them.* This point is now made in the discussion line 351-352.

On the genomics analysis, I have a few questions and requests for clarification.

- Fig 6. Why leave out 6 isolates? It seems many have identical core genomes based on this phylogeny. If there are sequence differences in these isolates that are hidden by the colors, then I'd ask for a different visualization that allows the reader to see genetic differences among the isolates.

*Six core genome sequences were shared by more than one isolates. In a distance tree, these occupy the same node. Thus, all sequences are present on the tree. Additionally, many of the branch lengths in this tree are very short, due to isolates that are separated by one or a small number of SNPs. We felt it was important to show the full phylogenic structure, including all samples, even though genetic differences between closely related isolates are not visible in this figure. These genetic differences are visualized in Fig 7. The Fig 6 legend has been revised to clarify this and states:*

‘Many isolates were closely related, resulting in very short branch lengths. See Fig 7 for more detailed visualization of phylogenetic structure within hosts.’

*.*

- Fig 7 (and S5 appendix). Please specify in the legend what it is we're looking at. This looks like a cladogram. Is it? What are the branch lengths? How was the tree generated? How is it rooted?

*The legend for Fig 7 has been revised to include the requested information. The legend now reads:*

A midpoint-rooted phylogeny was created based on the core genome alignment of all isolates using RAxML. Here, subsets of the full tree (Fig 6) are shown, including only the isolates from each patient (i.e. other tips are masked). Scale bars for branch length (mean number of substitutions per site) are included for each tree and insert. Rectangle inserts show detailed structures of indicated groups. Daptomycin MIC_C_s are listed for each tip. For tips representing two isolates with identical core genomes, the daptomycin MIC_C_s for both isolates are listed. Asterisks indicate mutations in genes known to be associated with variation in daptomycin susceptibility. For each patient, one assembled genome (two for the two clades in Patient 150) was used as a reference genome for variant calling (see Methods). A) Patient 4 B) Patient 86 C) Patient 150 D) Patient 87. Sequence data is available at NCBI GenBank under the study accession number PRJNA673360.

- Fig 7 key. "Infection" is not a timepoint. If the idea was to give a sense of temporal progression, could you please present a time-based tree?

*To allow a common legend and coloring across panels in figure 7, we used an ordinal time scale from earliest to latest swab sample, relative to the index sample. The infection (blood stream) samples were distinct in their sampling, and interpretation, so we did not place them on the same time scale. The infection happens to occur at the same time as the T-1 sample so we have added this to the legend to allow the sample to be placed within the temporal progression.*

- The amount of within-host diversity seems substantial, given the expected mutation rate of ~7 snps/genome / year.

*Indeed, we too were surprised at the substantial diversity within host, but this is not inconsistent with prior publications involving this organism (Moradigaravand, Genome Medicine 2017 and Dubin, Infection and Immunity 2019). The eco-evolutionary drivers of this diversity on short time scales and how they map to intermediate and long-term substitution rates we believe is an unresolved research question, one in which we are very actively involved. At this point, we have nothing to add beyond speculation which we do not feel adds much. Editorial advice welcome..*

- I'm confused as to the variant calling approach. Why were variants called against two references? If there were discrepancies, how was this dealt with?

*We have clarified our approach in the “Variant Calling” section of the Methods (from line 498). Briefly, reads from each sample were mapped against an assembled genome originating from the same patient. This allowed for variant calling between closely related samples. As an additional check to make sure we hadn’t missed potentially important variation in loci associated with daptomycin susceptibility, we mapped the reads from each sample against a set of loci from the E. faecium DO reference genome that had previously been associated with daptomycin susceptibility. The results from this second mapping were consistent with our first analysis, and no additional variants were added to our data set as a result of this analysis.*

- Please make clear which references and methods were used for generating each of the trees. It seems Fig 6 was made form a core genome alignment, but Fig 7 and S5 may have been from reference-based mapping?

*We have clarified these methods in the legend for each figure, and we have clarified the methods for variant calling. See the response to prior reviewer comments.*

- Tables in S5. I expect you are making all the assemblies available with the contigs and their annotations, since you're including contig number, position, and variant?

*Yes, the complete genomes with all contigs and PGAP annotations are available on* *NCBI GenBank. Accession: PRJNA673360*

- Please ensure that all sequencing data is available on NCBI GenBank.

*See above.*

Minor points.
- Suggest being careful with use of "after daptomycin", as it appears that some patients daptomycin use persisted through sampling. This includes some of the specimens with the highest levels of dapto resistance (they were obtained while the patient was on or had just been on daptomycin -- e.g., pt 86, pt 150).

*We appreciate the opportunity to clarify. In lines 221 and 226 we have changed ‘after the end of treatment’ to ‘after the index sample’ and ‘in the sample following the index sample’ as the reviewer is correct that in this context it may be misleading.*

- There are numerous typos (e.g., line 247 -- I think "150 cluster 1 and 150 cluster 2" is a typo, line 284 looks like it should be 'loss of vancomycin resistance' not 'loss of vancomycin susceptibility'). Suggest careful proofreading.

*Line 247 is not a typo but we have added ‘patient’ before the 150 to improve the clarity. The sentence now reads:*

‘By contrast, the mean number of core genome SNPs between two isolates within the remaining clusters was 4, 30, 2, 6, and 14 SNPs for patients 4, 86, 87, patient 150 cluster 1, and patient 150 cluster 2, respectively’

*Line 284 – we have made the suggested correction and have done a thorough proof reading of the entire manuscript.*
